# Novel assay to measure chromosome instability identifies *Punica granatum* extract that elevates CIN and has a potential for tumor- suppressing therapies

**DOI:** 10.3389/fbioe.2022.989932

**Published:** 2022-12-19

**Authors:** Nikolay V. Goncharov, Valeria A. Kovalskaia, Alexander O. Romanishin, Nikita A. Shved, Andrei S. Belousov, Vladlena S. Tiasto, Valeriia S. Gulaia, Vidushi S. Neergheen, Nawraj Rummun, Mikhail Liskovykh, Vladimir Larionov, Natalay Kouprina, Vadim V. Kumeiko

**Affiliations:** ^1^ A.V. Zhirmunsky National Scientific Center of Marine Biology, Russian Academy of Sciences, Vladivostok, Russia; ^2^ Institute of Life Sciences and Biomedicine, Far Eastern Federal University, Vladivostok, Russia; ^3^ Research Centre for Medical Genetics, Moscow, Russia; ^4^ School of Life Sciences, Immanuel Kant Baltic Federal University, Kaliningrad, Russia; ^5^ Biopharmaceutical Unit, Centre for Biomedical and Biomaterials Research (CBBR), University of Mauritius, Réduit, Mauritius; ^6^ Developmental Therapeutics Branch, National Cancer Institute, NIH, Bethesda, MD, United States

**Keywords:** chromosome instability, CIN, human artificial chromosome, HAC, *Punica granatum* leave extract, double-stranded breaks, micronuclei, natural products

## Abstract

Human artificial chromosomes (HACs) have provided a useful tool to study kinetochore structure and function, gene delivery, and gene expression. The HAC propagates and segregates properly in the cells. Recently, we have developed an experimental high-throughput imaging (HTI) HAC-based assay that allows the identification of genes whose depletion leads to chromosome instability (CIN). The HAC carries a *GFP* transgene that facilitates quantitative measurement of CIN. The loss of HAC/GFP may be measured by flow cytometry or fluorescence scanning microscope. Therefore, CIN rate can be measured by counting the proportion of fluorescent cells. Here, the HAC/GFP-based assay has been adapted to screen anticancer compounds for possible induction or elevation of CIN. We analyzed 24 cytotoxic plant extracts. *Punica granatum* leaf extract (PLE) indeed sharply increases CIN rate in HT1080 fibrosarcoma cells. PLE treatment leads to cell cycle arrest, reduction of mitotic index, and the increased numbers of micronuclei (MNi) and nucleoplasmic bridges (NPBs). PLE-mediated increased CIN correlates with the induction of double-stranded breaks (DSBs). We infer that the PLE extract contains a component(s) that elevate CIN, making it a candidate for further study as a potential cancer treatment. The data also provide a proof of principle for the utility of the HAC/GFP-based system in screening for natural products and other compounds that elevate CIN in cancer cells.

## Introduction

One of the most significant targets for emerging anticancer therapies is the transmission of chromosomes during cell division that involves a diverse number of regulatory elements, including components of the kinetochore complex and mitotic spindle associated proteins (Pérez de Castro and Malumbres, 2012). The disruption of these elements leads to chromosome instability (CIN), following the formation of aneuploid cells. As known, aneuploidy is a feature of most cancer cells and is often accompanied by an elevated rate of chromosome mis-segregation (Thompson et al., 2010). Gain or loss of entire chromosomes leads to large-scale changes in gene copy number and gene expression level (Kaufmann et al., 2014). Recent research data indicated that a high CIN rate in cancer cells prevents them from propagation and, thus, may be exploited therapeutically (Janssen et al., 2009; Silk et al., 2013).

In our previous publications, we developed quantitative assays for measuring CIN (Lee et al., 2013; Kim et al., 2016; Lee et al., 2016; Liskovykh et al., 2019). The assays are based on the use of a non-essential human artificial chromosome (HAC) carrying a constitutively expressed *GFP* transgene (HAC/GFP). This HAC propagates and segregates properly in fibrosarcoma cancer HT1080 cells. However, mitotic stability of HAC/GFP is approximately 10-fold lower than that of natural chromosomes (∼1 × 10-3) (Nakano et al., 2008; Iida et al., 2010), which makes the HAC a sensitive model for studying a CIN phenotype in cancer cells.

Two assays were used to rank different anticancer drugs with respect to their effects on chromosome transmission fidelity (Lee et al., 2013; Kim et al., 2016; Lee et al., 2016). Another HAC-based assay was applied for screening a siRNA library to identify unknown genes involved in CIN (Liskovykh et al., 2019). In all assays, the CIN rate is measured by flow cytometry, fluorescence microscopy, or a microplate reader. In general, these assays are based on the detection of loss of a HAC that carries a fluorescent reporter. The HACs contain a functional kinetochore and therefore are mitotically stable. However, the drugs that dysregulate proper segregation of native human chromosomes induce HAC mis-segregation which leads to the loss of a fluorescence signal. Therefore, the CIN rate can be measured by counting the proportion of fluorescent cells. Two HAC-based approaches utilize a GFP protein with a native half-life (Lee et al., 2013; Lee et al., 2016). The third HAC-based approach (Liskovykh et al., 2019) exploits expression of a modified short half-life *GFP* transgene (dGFP) that allows detection of CIN 72–120 h after its induction. In contrast, a standard *GFP* transgene significantly prolongs the experimental time up to 10–14 days and results in an accumulation of possible false-positive effects (Lee et al., 2013). The HAC/dGFP system was applied for the identification of human gene mutations in which lead to chromosome instability (Liskovykh et al., 2019) but was not applied for screening the drugs, treatment by which leads to the CIN phenotype in cancer cells.

The discovery of cancer drugs that are based on the screening of natural products continues to be an attractive approach in anticancer strategies (Neergheen-Bhujun et al., 2017). Natural biodiversity has been proven to be the prime source of bioactive molecules with a unique therapeutic spectrum, thus being the pillar of global healthcare (Kingston, 2011; Fridlender et al., 2015). Acquisition of samples from biodiversity-rich tropical forests, with emphasis on untapped endemic plants, was prioritized during the second phase of the National Cancer Institute (United States) drug screening program (Kinghorn, 1994).

In this study, we adapted the HAC/dGFP system to screen plant extracts with reported cytotoxic and cancer cell antiproliferative activities (Kiraz et al., 2016; Rummun et al., 2019; Rummun et al., 2020; Rummun et al., 2021a; Rummun et al., 2021b) for their ability to induce CIN in cancer cells. The HAC/dGFP-based assay identified *Punica granatum* leaf extract (PLE) which increases a CIN rate in HT1080 fibrosarcoma cells. We found that treatment of cancer cells with PLE leads to cell cycle arrest and formation of micronuclei (MNi) and nucleoplasmic bridges (NPBs). In addition, we observed that CIN mediated by PLE correlates with the induction of double-stranded breaks (DSBs). Thus, we demonstrate a potential of the HAC/dGFP-based assay for discovery of new anticancer drugs.

## Materials and methods

### Extracts

For the primary screening, we used a collection of extracts listed in Table 1. All the natural products were collected from Mauritius Island, exhaustively extracted, and dried as described in (Rummun et al., 2013; Rummun et al., 2019). The lyophilized powders of the extracts were dissolved in 100% DMSO to concentration 100 mg/ml. For screening, the LC_50_ concentrations were used (Table 1). The LC_50_ for PLE—a leader extract-was 190 μg/ml and was identified with the MTT assay. Extracts isolated from the wide range of plants were pre-selected, based on their explored anticancer properties (Rummun et al., 2013; Rummun et al., 2019).

**TABLE 1:** Natural products and their extracts used in this study.

*N*	Taxon	Family	Herbarium voucher number	Ethno medicinal uses Rummun et al. (2013); Rummun et al. (2018)	LC_50_ HT1080 HAC/dGFP mg/ml
1	*Acalypha integrifolia*	Euphorbiaceae	MAU 0016402	Anti-helminthic, astringent, depurative, purgative, skin infections, tambav	56
2	*Diospyros chrysophyllos Poir*	Ebenaceae	MAU 0009431	Not documented	99
3	*Diospyros egrettarum I.B.K. Richardson*	Ebenaceae	MAU 0018728	Not documented	142
4	*Diospyros leucomelas Poir*	Ebenaceae	MAU 0016547	Not documented	157
5	*Diospyros tessellaria Poir*	Ebenaceae	MAU 0016639	Not documented	172
6	*Dombeya acutangula Cav. subsp. rosea Friedmann*	Malvaceae	MAU 0016638	Astringent, control blood flow, dysentery	56
7	*Eugenia lucida Lam*	Myrtaceae	MAU 0016552	Not documented	88
8	*Eugenia orbiculata Lam*	Myrtaceae	MAU 0002703	Not documented	117
9	*Eugenia pollicina Guého & A. J. Scott*	Myrtaceae	MAU 0017468	Not documented	150
10	*Eugenia tinifolia Lam*	Myrtaceae	MAU 0016540	Depurative, skin diseases	75
11	*Hancea integrifolia (Willd.) S.E.C. Sierra, - Kulju & Welzen*	Euphorbiaceae	MAU 0016431	Clean the blood and improve blood circulation, tonic	100
12	*Labourdonnaisia glauca Bojer*	Sapotaceae	MAU 0016430	Emmenagogues	192
13	*Mimusops petiolaris (DC.) Dubard*	Sapotaceae	MAU 0016640	Astringent, diarrhoea, dysentery, haemorrhage	146
14	*Sideroxylon boutonianum A.DC*	Sapotaceae	MAU 0016546	Not documented	96
15	*Sideroxylon cinereum Lam*	Sapotaceae	MAU 0016429	Not documented	104
16	*Sideroxylon sessiliflorum (Poir.) Capuron ex Aubréville*	Sapotaceae	MAU 0009459	Not documented	133
17	*Stillingia lineata Muell. Arg*	Euphorbiaceae	MAU 0016545	Eczema, skin disease	157
18	*Terminalia bentzoë (L.) L.f. subsp. Bentzoë*	Combretaceae	MAU 0016557	Asthma, antipyretic, antimalarial, chills, dysentery, diarrhoea, depurative, emmenagogue, haemorrhages, Sexually transmisible diseases	136
19	*Syzygium bijouxii Guého et A. J. Scott*	Myrtaceae	MAU 0013805	Not documented	129
20	*Syzygium coriaceum Bosser* and *Guého*	Myrtaceae	MAU 0016404	Not documented	140
21	*Syzygium guehoi Bosser* and *D. Florens*	Myrtaceae	MAU 0016549	Not documented	142
22	*Syzygium pyneei Byng, V.Florens* and *Baider*	Myrtaceae	MAU 0014026	Not documented	110
23	*Syzygium glomeratum*	Myrtaceae	MAU 0016432	Cough, headache	102
24	*Punica granatum L.*	Punicaceae	MAU 0016480	Asthma, diarrhea, dysentery, anti-helminthic	190

### Cell lines and culture conditions

The following cell lines were used for all the experiments described below: the pigmented epithelium immortalized cells RPE-1 (hTERT RPE1-1) and the human fibrosarcoma cell line HT1080 (ATCC^®^ CCL-121™). The HT1080 and RPE-1 cell lines were obtained from the American Type Culture Collection and were authenticated both morphologically and by short tandem repeat analysis. The cell lines were tested regularly to confirm the lack of mycoplasma infection with the mycoplasma detection kit PlasmoTest (InvivoGen, United States). The HT1080 and RPE-1 cells were basically maintained in Dulbecco’s Modified Eagle’s Medium (DMEM) (Gibco, United States) supplemented with 10% (v/v) fetal bovine serum (Clontech Laboratories, Inc.) at 37°C in 5% CO_2_ atmosphere. In order to achieve the selection for the GFP-positive cells, HT1080 cells carrying the HAC/dGFP were maintained in the presence of 10 μg/ml of Blasticidin S (Funakoshi Inc., Japan).

### Hamilton microlab star liquid handling workstation

Cell seeding, adding all liquid substances such as culture medium, extract samples, staining and fixation solutions, aliquoting and distribution were performed on the Hamilton Microlab Star Liquid Handling Workstation (United States). For Hamilton Microlab Star Liquid Handling Workstation experiments, 10 mM HEPES (Sigma Aldrich, United States) was additionally added to culture media to maintain proper pH out of CO_2_ enriched atmosphere. The pipetting array consisted of 4 pipetting heads and used 1,000 and 50 μl compressed O-ring expansion (CO-RE) tips. All liquids were aspirated at a fixed height from the container bottom (0.5 mm) and dispensed using the jet empty setting. The aspiration and dispensation rate in a cell culture containing wells was set to 10 μl/s. Cells and samples were mixed (consisting of aspiration and dispense of 1,000 μl of liquid into the same container) 3 times prior to being transferred to the incubation plate to ensure even cell distribution and sample uniformity. Cell culture incubation was performed using the Hamilton Heater Shaker module. To reduce microbial contamination, prior to executing the protocol, a fully loaded workstation was decontaminated using an external UV light source. All the reagents were contained in 60- and 120-ml Hamilton Reagent Reservoirs covered with tinfoil (later pierced by a pipetting head).

### LC_50_ determination using MTT assay

HT1080 cells were seeded in a 96-well plate (Eppendorf, Germany) at density of 1 × 10^3^ cells per well and were kept at 37°C under 5% CO_2_. 24 h later, the cells were treated with extracts at concentrations of 50, 100, 150, 200, and 250 μg/ml. 24 h later, the medium was discarded and replaced by 100 μl of DMEM, 10% FBS containing 5 mg/ml of MTT (3-(4, 5-Dimethylthiazol-2-yl)-2, 5-diphenyltetrazolium bromide) (Sigma, Germany). After 4 h of incubation, 100 μl of lysis buffer (4 mM HCL, 1% Np40 in isopropanol) was added and the cells were kept for 20 min on shaker. Using micro-plate reader iMark (Bio-Rad, United States), absorbance of each well was measured in 595 and 655 nm. The experiment was done in 6 replicates for each sample. GraphPad Prism software (San-Diego, United States) was used for statistical analysis. The obtained data were normalized for the percentages (a negative control data was considered as 100%) in order to calculate LC_50_. Linear-regression analysis was performed where *R* ^2^ greater than 0.65 was considered relevant.

### Calculation of human artificial chromosomes loss after extract treatment

The HAC stably propagates in human HT1080 cells, i.e., almost every cell contains one copy of the HAC. The HAC is less stable than the host chromosomes (Nakano et al., 2008). Therefore, the loss of HAC/GFP can be measured by flow cytometry or fluorescence scanning microscope. The HAC-loss is measured without selection when the cells start losing the HAC spontaneously. To measure HAC loss, we showed the ratio of GFP-negative cells after treatment with extracts. Induction of the chromosome instability due to exposure to the natural extracts was examined in human cancer HT1080 cells carrying the HAC/dGFP. For the experiment, cells were seeded and cultured with 10 μg/ml of Blasticidin S. After a day, the seeding medium was removed, then replaced by a new portion of the culture medium containing LC_50_ concentrations of the natural extracts (Supplementary Figure S1) and free of Blasticidin S and left for 24 h incubation. After 24 h, the extract-containing medium was replaced with a fresh medium without the extract and free of Blasticidin S. The cells were subsequently grown without blasticidin selection for 72 h, then HT1080 cells were analyzed for HAC loss by detecting the ratio of GFP-negative cells measured independently using Synergy™ HTX Multi-Mode Microplate Reader (BioTek, United States) and BD Accuri C6 (BD Biosciences, United States). As a positive control, we used the anti-mitotic, spindle-targeting compound Taxol at concentration 6 nM (Supplementary Figure S1). At this concentration Taxol induces micronuclei (MNi) formation and chromosome loss. As a negative control, the cells were treated with 0.2% DMSO. For each extract, the experiment was carried out in 3 replicates. The rate of HAC loss after cell treatment by the extract can be also calculated using a formular described in Supplementary Material.

### Visualization of human artificial chromosomes loss with microplate reader and flowcytometry

For each extract, the rate of HAC/GFP loss was accurately measured independently using Synergy™ HTX Multi-Mode Microplate Reader (BioTek, United States) and BD Accuri C6 (BD Biosciences, United States) for a double check. For Synergy™ HTX Multi-Mode Microplate Reader (BioTek,United States), 1 × 10^5^ cells were seeded on a 96-well plate and depicted 24 h after extract treatment under 4х magnification in 5 × 5 fields of view. The number of GFP-positive and GFP-negative cells were counted in each field with threshold 7,000. The cell size was restricted from 5 μm up to 100 μm. For flowcytometry experiment using BD Accuri C6 (BD Biosciences, United States), 4 × 10^5^ cells per well were seeded on a 24-well plate with 500 μl of the culture medium and 10 μg/ml of Blasticidin S. Then cells were harvested by trypsin-treatment and resuspended in PBS containing 3 µM of DRUQ7. All the samples were vortexed immediately prior to flow cytometry examination using BD Accuri C6 (BD Biosciences, United States), the Fluorescence of GFP-positive cells was measured by the 488 nm laser and detected at 510 nm. The death cells were counted by DRUQ7 fluorescence excited by the 640 nm laser and detected at 722 nm. Samples were acquired in at least three separate triplicates for 30 s or 1.5 × 104 events (at minimum).

### Statistical analysis

The statistical significance of comparisons was determined by GraphPad Prism 6. An unpaired Student *T*-test and One-way Annova with multiple comparisons were used. *p* < 0.05 was considered significant. The data are presented in diagrams as mean, error bars correspond to 95% confidence interval.

### Measurement of cell proliferation using a high content imaging system (cell IQ^®^)

After treatment with PLE, its effect on cell proliferation was measured. HT1080 and RPE1-1 cells were seeded in Greiner Bio-One TC-plate 24-well plates, 5,000 cells per well. The next day after attachment and cell spreading, PLE was added to the culture medium at LC_50_ concentrations. 0.2% DMSO was used as a negative control and 6 nM of Taxol as a positive control. At 24 h after PLE treatment, the medium was replaced with a fresh complete DMEM. During 114 h, attached cells were observed using the automated cell culture and analysis system Cell-IQ^®^ (CM Technologies) with Nikon CFI Plan Fluorescence DL ×4 objective. Nine visual fields were analyzed in each well. The interval between observations of each visual field was 3 h. Cell counting and cell growth curves were obtained using Cell-IQ Analyzer ™ software.

### Flow cytometric analysis of cell cycle using Hoechst 33342 DNA staining

HT1080 and RPE-1 cells were cultured in a 24 well-plate (Eppendorf, Germany). Cells were treated with PLE at LC_50_ concentration for 24 h. 0.2% of DMSO was used as a negative control (untreated cells). The flow cytometric analysis was performed 24 and 72 h after PLE treatment. To perform the flow cytometry, the cells were collected in microcentrifuge tube and fixed with 4% PFA in PBS for 10 min, then centrifuged for 10 min at 150 g and permeabilizated with 500 μl of 100% methanol on ice. Then 1 μg/ml of Hoechst 33342 solution in PBS was prepared.106 cells in 1 ml of Hoechst 33342 solution, wrapped with aluminum foil to avoid direct light, was resuspended and incubated at room temperature for 60 min. All the samples were vortexed immediately before flow cytometry examination. Fluorescence of Hoechst 33342 positive cells was measured by emission 355 nm laser with extension 448 nm. Samples were acquired in at least three separate triplicates for 30 s or 1.0 × 10^4^ events. Flow cytometry was performed using C-Flow BC (moFlo Astrios EQ) and analyzed in Kaluza, Flow Cytometry Analysis Software - Beckman Coulter. Cell cycle analysis was performed with MoFlo Estrios EQ cell sorter (Beckman Coulter, United States) and software Kaluza Analysis (v 3.1).

### Mitotic index analysis with fluorescence microscopy

Mitotic index was determined for HT1080 and RPE-1 cells 24 and 72 h after PLE treatment. HT1080 and RPE-1 cells were cultured in DMEM supplemented with 10% FBS. The medium was aspirated, and the plate was washed with PBS once. Cells were fixed by 4% formaldehyde solution in PBS. 1 × 10^5^ HT1080 and RPE-1 cells per well were plated on ibidi-cell culture dishes (Ibidi, United States) and treated with PLE extract at LC_50_ concentration for 24 h. Cells were fixed with 3.7% formaldehyde in PBS for 10 min at room temperature, then permeabilized with 0.2% Triton X-100 in PBS for 10 min. Cells were blocked with 5% milk in TBS for 2 h at room temperature and treated with cytoskeletal β-Tubulin rabbit monoclonal antibody (Abcam, United States) in 1:1,000 dilution with 5% milk/TBS at 4°C overnight. Goat Anti-Rabbit Alexa Fluor^®^ 568 conjugated secondary antibodies were used in 1:500 dilution with 5% milk/TBS at room temperature for 2 h. Then cells were stained with DAPI (diluted 1:15,000 in PBS) for 10 min at room temperature. Quantification was performed using the FV1200 confocal laser scanning microscopy system equipped with the objective lens (UPLSAPO ×100) (Olympus, Japan). A 405 nm LD Laser with Integrated Transmitted Light Photomultiplier Detector and 488 nm Argon Laser with High-Sensitivity Detector (GaAsP) were used. To avoid cross detection, the images were acquired sequentially at 488 nm (Argon) and 405 nm (LD). All taken Z-sections were condensed into one single plane in order to visualize all detectable microtubules and chromosomes.

### Staining and quantification of γ-H2AX foci

1 × 10^5^ HT1080 and RPE-1 cells per well were plated on ibidi-cell culture dishes (Ibidi, United States) and treated with PLE extract at LC_50_ concentration for 24 h. As a positive control, Etoposide was used at concentration 1 μM. Cells were fixed with 3.7% formaldehyde in PBS for 10 min at room temperature, then permeabilized with 0.2% Triton X-100 in PBS for 10 min. Cells were blocked with 5% milk in TBS for 2 h at room temperature and treated with rabbit monoclonal anti-phospho γ-H2AX (Ser139) antibody (Abcam, United States) in 1:500 dilution with 5% milk/TBS at 4°C overnight. Goat Anti-Rabbit Alexa Fluor^®^ 568 conjugated secondary antibodies were used in 1:500 dilution with 5% milk/TBS at room temperature for 2 h. Cells were stained with DAPI (diluted 1:15,000 in PBS) for 10 min at room temperature. Quantification of the γ-H2AX foci in nuclear was performed using the FV1200 confocal laser scanning microscopy system equipped with the objective lens (UPLSAPO ×100) (Olympus, Japan). A 405 nm LD Laser with Integrated Transmitted Light Photomultiplier Detector and 488 nm Argon Laser with High-Sensitivity Detector (GaAsP) were used. To avoid cross detection, the images were acquired sequentially at 488 nm (Argon) and 405 nm (LD). All taken Z-sections were condensed into one single plane in order to visualize all detectable foci.

### Cytokinesis-block micronucleus assay and nucleoplasmic bridges formation

Cytokinesis-block micronucleus formation assay was performed as described earlier (Fenech 2007) with minor changes: 1 × 10^5^ HT1080 or RPE-1 cells per well were plated on ibidi-cell culture dishes (ibidi, United States) and treated with PLE extract at LC_50_ concentration for 24 h. Cells were fixed with 3.7% formaldehyde in PBS for 10 min at room temperature. Scoring Procedure Cells were stained using 15 μl of a DNA-specific stain, namely 40,6-diamino-2-phenylindole dihydrochloride (DAPI). About 2,000 cells were scored using the FV1200 confocal laser scanning microscopy system equipped with the objective lens (UPLSAPO ×100) (Olympus, Japan) and 405 nm LD Laser with Integrated Transmitted Light Photomultiplier Detector. The criteria for evaluation of micronucleus formation assay were: 1) cell integrity (intact nucleus and cytoplasm); 2) similar staining of nucleus and MNi; 3) nucleus and MNi are in the same plate. The frequency of cells with micronuclei (MNi) and nucleoplasmic bridges (NPBs) was determined for each analyzed subject.

### Analysis of apoptosis

5 × 10^5^ HT1080 and RPE-1 cells were seeded in 24 well-plates. Next, the PLE extract at LC_50_ concentration was added to both cells. 24 and 72 h after PLE treatment, cells were trypsinized and diluted in DMEM. Then Flowcytometry analysis was performed using MoFlo Astrios EQ cell sorter (Beckman Coulter, United States). Comparison of early apoptotic and necrotic cells 24 and 72 h after PLE treatment was performed with propidium iodide and annexin V-FITC. Analysis of the flowcytometry data was performed using Kaluza Software v2.1 (Beckman Coulter, United States). All the samples had three technical replicates; statistical analysis was done using One-way ANOVA (Dunnet correction) *via* Prizm 8.0.0 (GraphPad Software, United States).

## Results

### Experimental system for screening anticancer drugs that elevate chromosome instability

**FIGURE 1:**
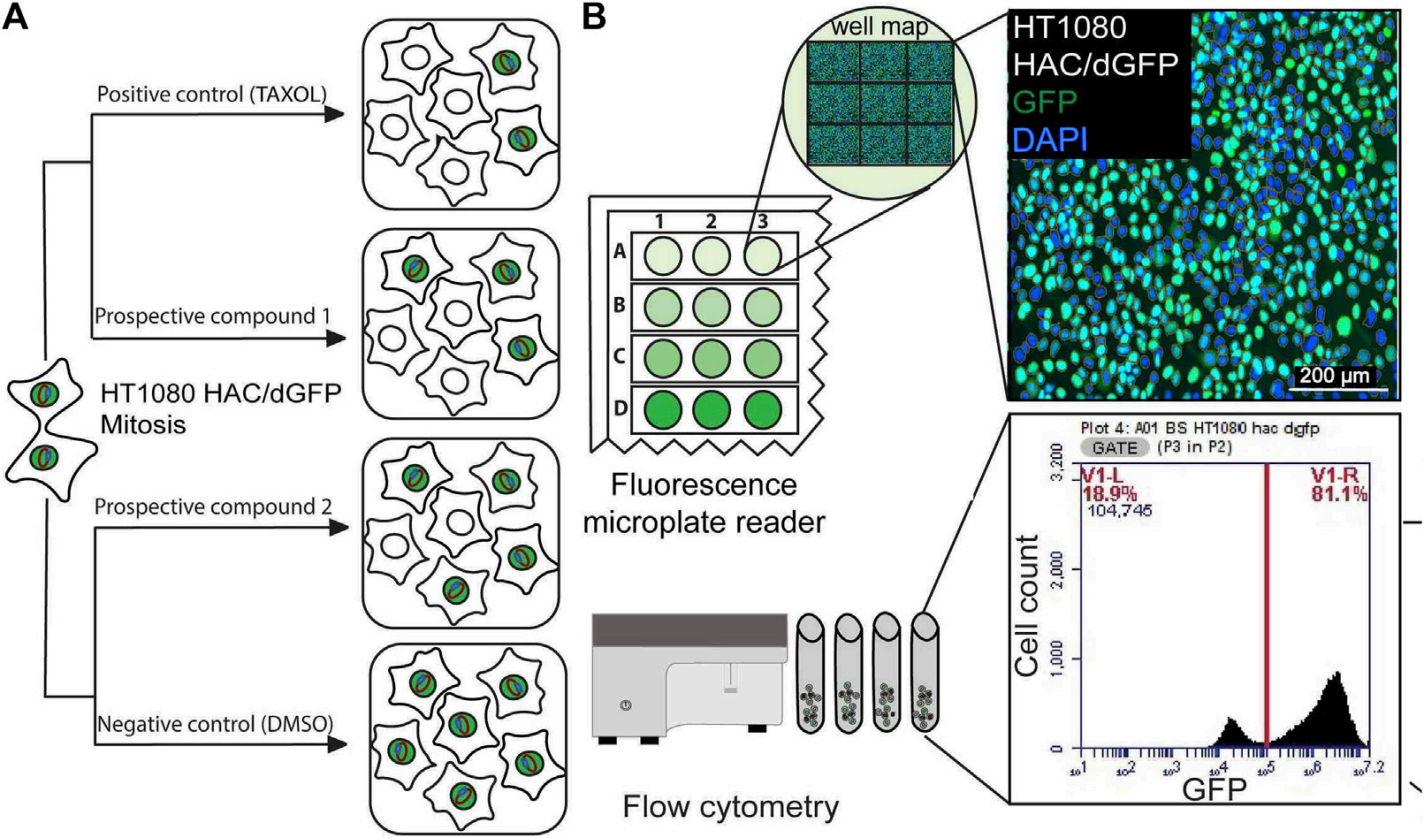
**(A)** A scheme of the HAC/dGFP assay. The assay is based on the use of the HAC/dGFP (the alphoid^tetO^-HAC containing the dGFP transgene). Cells with the HAC display green fluorescence, while cells that lack it do not. It is expected that untreated cells (“a negative control”) should show uniform green fluorescence. **(B)** A cell population, that has lost HAC upon treatment with the CIN-inducing drug (“a prospective compound”), loses GFP fluorescence. For the positive control, we used Taxol, a CIN-inducing microtubule destabilizing agent. The actual number and percentage of cells with the HAC/dGFP are measured by a fluorescence microplate reader and flow cytometry.

In this study, we adapted the HAC/dGFP system (Liskovykh et al., 2019) for drug screening using Synergy™ HTX Multi-Mode Microplate Reader that is used for fluorescence detection. The HAC/dGFP system is based on the use of the alphoid^tetO^-HAC (Nakano et al., 2008) containing the dGFP transgene, where CIN detection can be identified by loss of fluorescence (Figure 1). The alphoid^tetO^-HAC propagated in the HT1080 human fibrosarcoma cells are sensitive to the drug treatment and other factors affecting chromosome transmission. The HAC/dGFP carries a new class of the GFP-fusion, i.e., a “destabilized GFP” (dGFP). The first approach that utilized a destabilized GFP was described by Sakaue-Sawano et al. (2008) and Sakaue-Sawano et al. (2013). This method is based on the expression of short FUCCI (fluorescent ubiquitination-based cell-cycle indicator) peptides CDT1 and GMNN in one reading frame with fluorescent protein coding sequences. Expression of these fusions may be visualized at the different stages of cell cycle. The HAC/dGFP expresses two fusions of GFP: GFP-fused with a 30–120 amino acid domain of CDT1, and GFP-fused with a 1–110 amino acid domain of DNA replication inhibitor (GMNN). FUCCI domains lead to proteasomal degradation of GFP proteins associated with their C-terminal sequences, and the time of active functioning of each is strictly related to the duration of different phases of cell cycle. CDT1 is expressed during the G1 period, while GEMININ is expressed during the S, G2, and M periods. Therefore, the GFP-CDT1 fusion will cause the HAC-containing cells to be green in G1 phase of the cell cycle. The GFP-GMNN fusion causes the HAC-containing cells to be green in S-G2-M phases of the cell cycle. Thus, the cells carrying the HAC/dGFP show a robust fluorescent signal throughout the cell cycle and lose the GFP signal within hours after HAC loss (Liskovykh et al., 2019).

The rate of HAC loss driven by drug treatment can be measured by loss of a fluorescent signal. Cells with the HAC/dGFP display green fluorescence, while cells that lack it do not. It is expected that untreated cells (“a negative control”) should show uniform green fluorescence. A cell population that has lost HAC upon treatment with the CIN-inducing drugs (“prospective compounds”) should be highly variable in fluorescence (Figure 1A).

Our previously published screening of a siRNA library of protein kinases using HT1080 cells carrying the HAC/dGFP was performed using Laser Scanning Microscope. In this work, we detected a ratio of GFP-negative cells using a fluorescent microplate reader and flow cytometry (Figure 1B). Moreover, all the procedures from cell seeding to drug treatment and fixation were performed on 96-well plates automatically on the Microlab Star liquid handling workstation (Hamilton, United States), which ensured consistency across replicates. As a positive control, we used Taxol, a CIN-inducing microtubule destabilizing agent. As shown earlier, Taxol exhibits the highest destabilizing effect on the HAC and natural chromosomes (Lee et al., 2016). We demonstrated the capability of the HAC/dGFP system to identify substances that induce CIN over 72 h.

### Screening of natural products and their extracts revealed that the *P. granatum* leaves extract has a significant effect on the human artificial chromosomes mis-segregation rate during mitotic divisions

**FIGURE 2:**
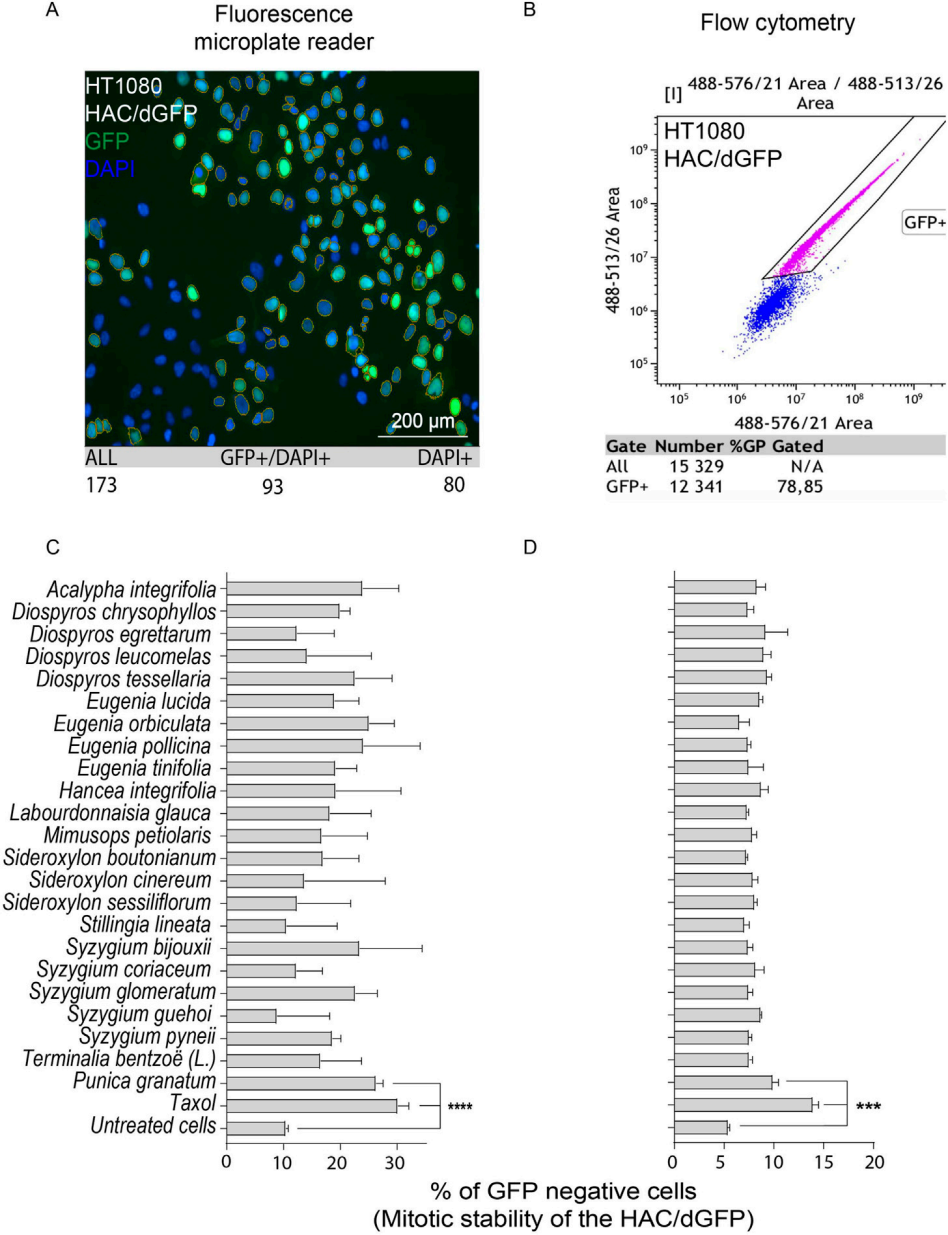
Mitotic stability of the HAC/dGFP in human cancer HT1080 cells treated with the natural extracts. Mitotic stability was measured by a fluorescent microplate reader **(A,C)** and flow cytometry **(B,D)**. Among 24 extracts analyzed, the strongest effect on the HAC/dGFP stability was demonstrated by the cells treated with the *Punica granatum* leaves extract (PLE). Asterisks indicate statistical significance above negative control (*T*-test, *p* < 0.01).

For primary screening, we chose the collection of natural extracts from tropical forest plants that have been used in traditional medicine. Their cytotoxic activity was demonstrated in many cancer cell lines (Kiraz et al., 2016; Rummun et al., 2019; Rummun et al., 2020; Rummun et al., 2021a; Rummun et al., 2021b). For each extract used in this study, we determined the LC_50_ concentration, i.e., the conditions under which the viability of cells would be around 50%, using the MTT assay adapted for human cancer HT1080 cells carrying the HAC/dGFP (Supplementary Figure S1). All LC_50_ concentrations are listed in Table 1. Those concentrations were used for induction of CIN (a HAC-loss; see MATERIALS and METHODS). None of the compounds, except the *P. granatum* leaves extract (PLE), showed a significant effect on HAC/dGFP stability by either fluorescence microplate reader (Figures 2A,C) or flow cytometry assays (Figures 2B,D). Based on the data obtained by flow cytometry, the average ratio of GFP-negative cells after PLE treatment is 9.916 (SD ± 0.6402; 1.83-fold elevation compared to the negative control (untreated cells) and 1.4-fold decrease compared to the positive control (Taxol-treated cells). Based on the data obtained by microplate reader, the average ratio of GFP negative cells after PLE treatment is 26.28 (SD ± 1.237; 2.53-fold elevation compared to the negative control and 1.15-fold decrease compared to the positive control. To exclude the possibility of a false-positive result, we measured the accumulation of GFP-negative cells 3 days after PLE treatment. The effect of HAC/dGFP instability intensified upon two divisions 24 h after PLE treatment and reached a maximum on day 4 (Supplementary Figure S2).

Thus, the HAC/dGFP-based assay allowed us to rank 24 natural products and their extracts with respect to their effect on chromosome stability. The *P. granatum* leaves extract exhibited the highest effect on HAC/dGFP stability and, therefore, may be considered as a promising candidate for future drug development.

### *P. granatum* leaf extract treatment leads to cell cycle arrest and reduction of mitotic index

**FIGURE 3:**
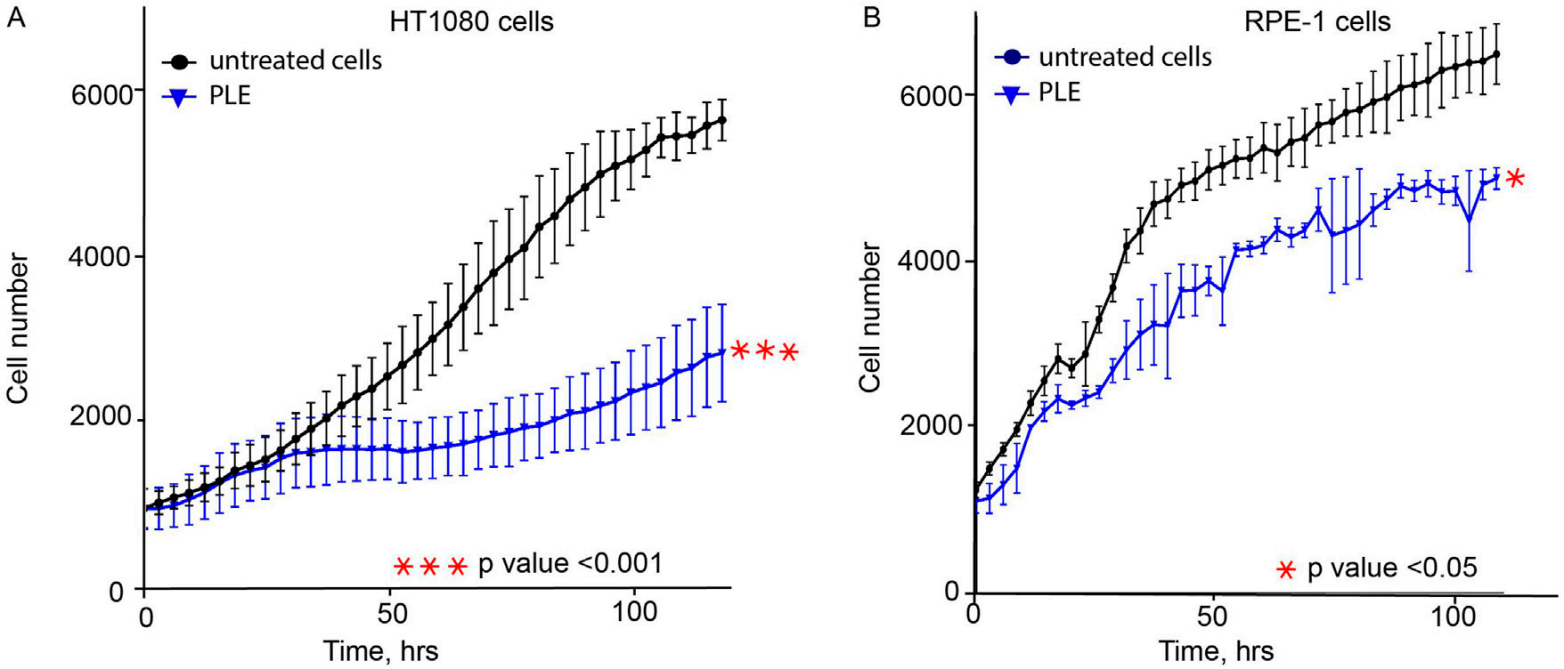
The proliferation rate of HT1080 **(A)** and RPE-1 **(B)** cells treated with PLE. Cells were treated for 24 h, then media was replaced, and cell proliferation was recorded by live imaging for 120 h. PLE—*P. granatum* leaves. Each cell line was compared to a negative control: HT1080 PLE-treated cells compared to HT1080 untreated cells; RPE-1 PLE-treated cells compared to RPE-1 untreated cells. The statistical analysis was performed using an unpaired Student's t-test. Asterisks indicate statistical significance (*p < 0.05, ***p < 0.001, relative to the negative control – untreated cells).

An additional set of experiments was performed to observe other CIN-related phenotypes in PLE-treated cells that proves the ability of the HAC/dGFP-based approach to detect CIN mediated by drug treatment. To rule out the cancer cell line-specific phenotype of HT1080, the proliferation, cell cycle, and mitotic progression assays have been also performed with human retinal pigmented epithelial-1 (RPE-1) cells immortalized by the TERT1 transgene. RPE-1 cells are increasingly being used as a model to study mitosis because they represent non-transformed cells alternative to cancer cell lines, such as HT1080 or HeLa. First, we determined the LC_50_ concentration of PLE in RPE-1 cells (Supplementary Figure S1). According to the data from cell tracking microscopy (Cell IQ), PLE at LC_50_ concentration for HT1080 and RPE-1 cells inhibits cell proliferation (Figures 3A,B). We have observed that PLE treatment decreased the rate of proliferation in both RPE-1 and HT1080 cells relative to untreated cells. In RPE-1 cells, we found that proliferation rates decreased 1.22, 1.36, 1.25, 1.26, and 1.44-fold when measured 24, 48, 72, 96, and 144 h after treatment, respectively. Similarly, proliferation rates in HT1080 cells decreased 1.11, 1.51, 2.09, 2.18, and 1.98 -fold when measured 24, 48, 72, 96, and 114 h after PLE treatment.

**FIGURE 4:**
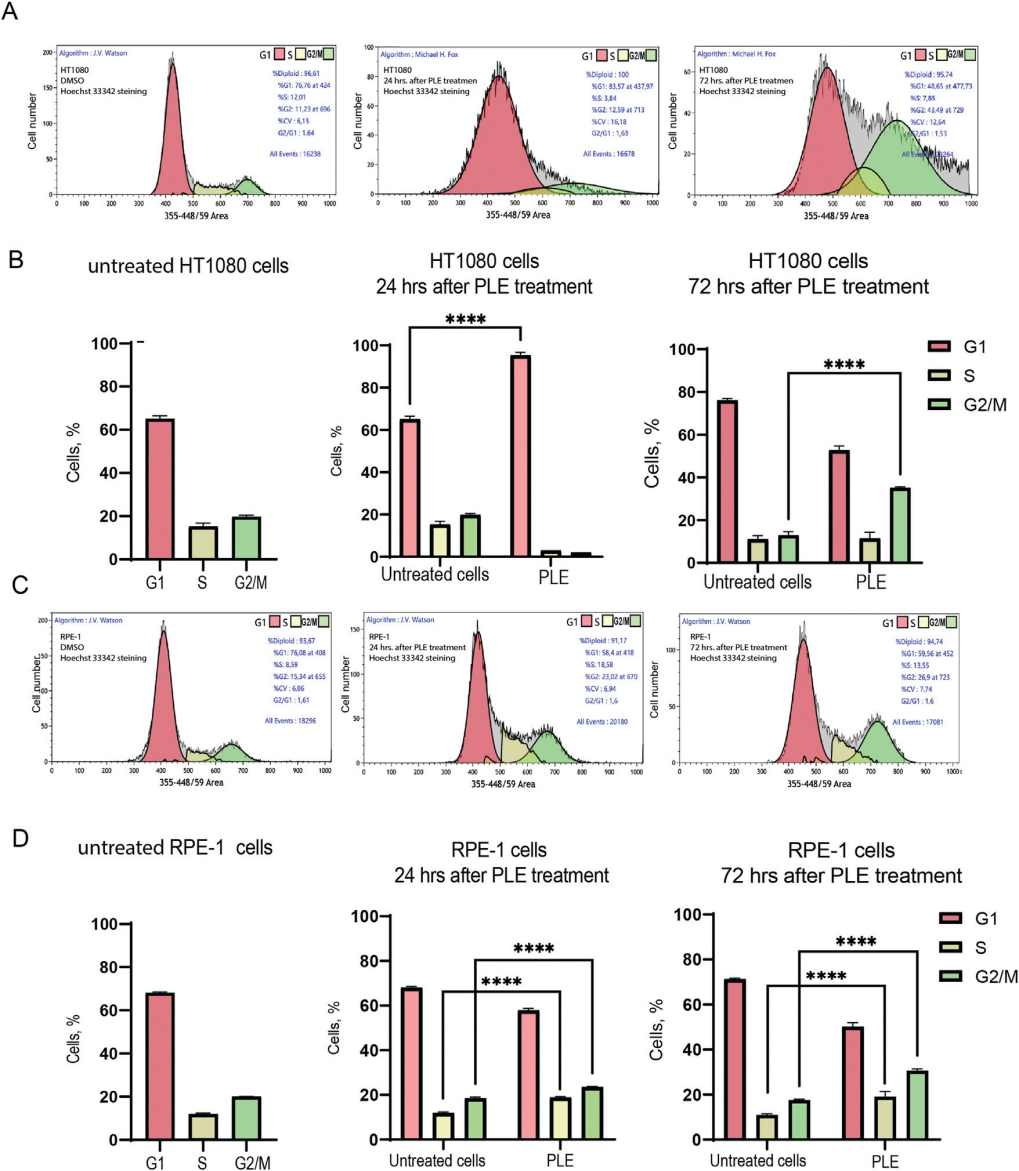
Analysis of the cell cycle after PLE treatment. **(A)** Flow cytometry analysis of HT1080 untreated cells 24 and 72 h after PLE treatment. **(B)** The percentage of cells in G1, S and G2/M phases in HT1080 cell population after 24 and 72 h. **(C)** Flow cytometry analysis of RPE-1 untreated cells after 24 and 72 h. **(D)** The percentage of cells in G1, S and G2/M phases in RPE-1 cell population after 24 and 72 h. The statistical analysis was performed using 2way ANNOVA, asterisks indicate statistical significance (****p < 0,0001, relative to the negative control—untreated cells).

To determine possible causes of inhibition of cell proliferation, we performed a cell cycle analysis of HT1080 and RPE-1 cells 24 and 72 h after PLE treatment (Figure 4; Supplementary Table S1). Flow cytofluorometry data showed accumulation of HT1080 cells in G1 phase after 24 h. The average percentage of cells was 1.46-fold elevated compared to the negative control (Figure 4B). After 72 h, cells accumulated in G2/M, 2.72-fold elevation (Figure 4B). RPE-1 cells accumulated in G2/M phase after 24 h, 1.27-fold elevation, and after 72 h, 1.74-fold elevation (Figure 4D). Moreover RPE-1 cells accumulated in S the phase after 24 h, 1.59-fold elevation, and after 72 h, 1.73-fold elevation (Figure 4D). The fact that PLE treatment leads to stronger G1 delay in cancer HT1080 compared to RPE-1 cells may be explained by alterations in the genes involved in G1 checkpoint control that are the most frequent changes detected in cancer cells (Ahmed et al., 2022). Cell cycle delays at S and G2/M phase observed in RPE-1 cells may result from activation of the DNA damage checkpoint pathway (Gomez and Hergovich, 2016; Lezaja and Altmeyer, 2018).

**FIGURE 5:**
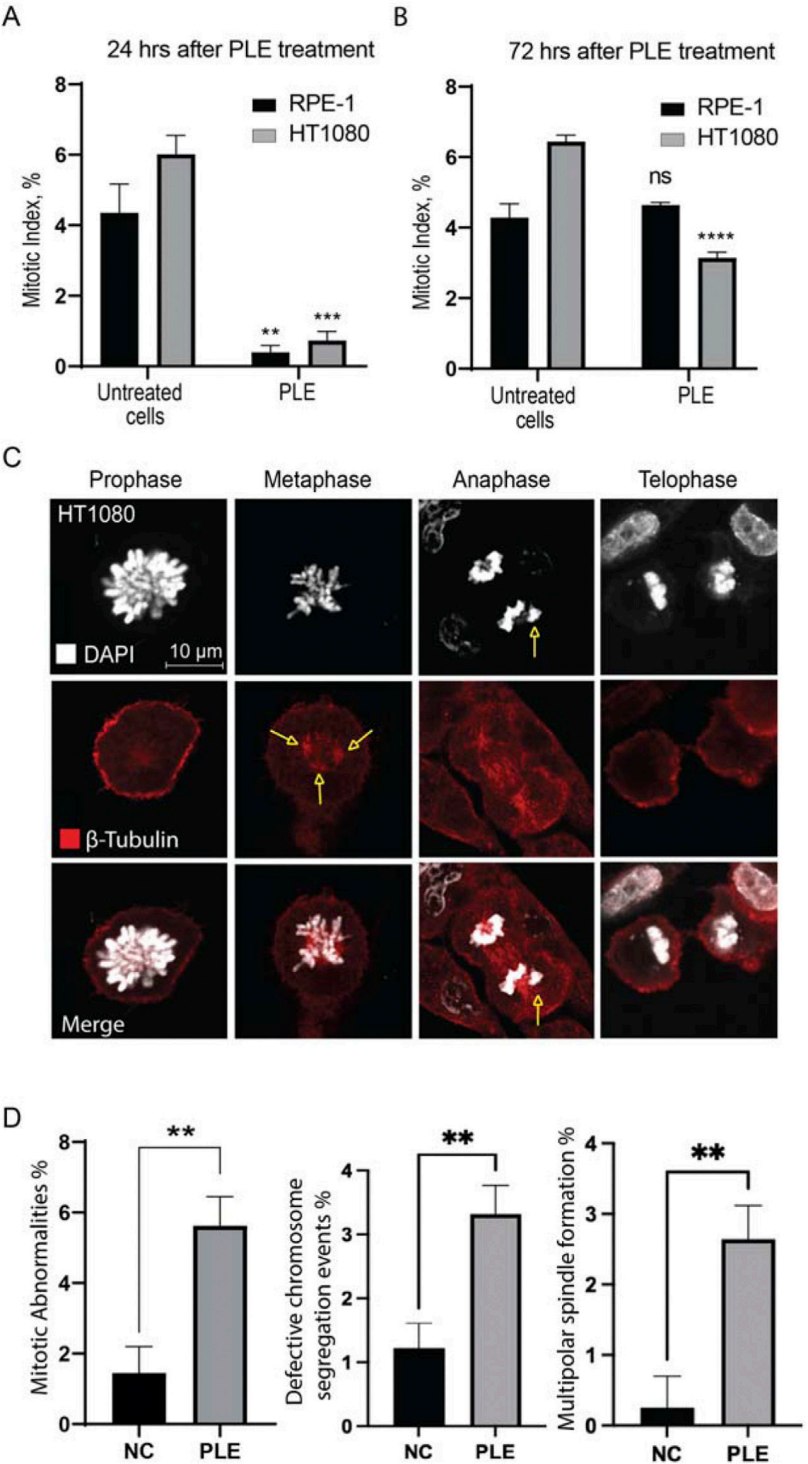
Mitotic index of HT1080 and RPE-1 cells treated with PLE. **(A)** The percentage of mitosis in HT1080 and RPE-1cells 24 h after PLE treatment. **(B)** The percentage of mitosis 72 h after PLE treatment. **(C)** Analysis of localization of tubulin beta at the different stages of mitosis after PLE treatment in HT1080 cells. Staining by antibodies against tubulin beta is marked in red and DAPI in white. Yellow arrows point to the observed mitotic abnormalities. **(D)** Proportion of observed mitotic abnormalities in HT1080 cells. Representative pictures of localization of tubulin beta at the different stages of mitosis in untreated HT1080 cells are shown in Supplementary Figure S4. Between 100 and 150 mitotic events were analyzed. Statistical analysis was performed using 2way ANNOVA, asterisks indicate statistical significance (**p < 0.01, ***p < 0.001, ****p < 0.0001 relative to the negative control—untreated cells).

The mitotic index (the ratio of the number of cells in a population undergoing mitosis to the total number of cells) analysis of RPE-1 and HT1080 cells after PLE treatment supports the cell cycle data. We observed a statistically significant decrease of the mitotic index in both RPE-1 and HT1080 cells 24 h after PLE treatment The average value of the mitotic index of HT1080 cells was 0.732,908 (SD ± 0.2587; 8.21-fold decrease) and RPE-1 cells was 0.399,191 (SD ± 0.1931; 10.91-fold decrease) (Figure 5A; Supplementary Table S2). After 72 h, the mitotic index was significantly decreased only in HT1080 cells (Figure 5B; Supplementary Table S2). The average value of the mitotic index was 3.14054 (SD ± 0.1615; 2.05-fold decrease). An apoptotic assay revealed a significant induction of early apoptosis in HT1080 cells 24 and 72 h after PLE treatment (Supplementary Figure S3). The apoptosis-dependent cell elimination perhaps caused a significant decrease in mitotic index.

Reduction of mitotic index and cell cycle arrest indicate to the presence of mitotic abnormalities. Therefore, next, we characterized in more detail the mitotic defects at the different stages of mitosis observed after PLE treatment in HT1080 cells. The cells were stained with antibodies against β-tubulin to visualize the mitotic spindles and DAPI to visualize chromatin. PLE treatment of HT1080 cells revealed a significant increase in mitotic abnormalities that included defects in chromosome alignment in anaphase and presence of multipolar spindles in metaphase (Figures 5C,D; Supplementary Table S5). To summarize, mitotic abnormalities observed after PLE treatment can lead to destabilization of chromosomes, aneuploidy, chromosome damage, and micronucleus formation.

### *P. granatum* leaf extract treatment leads to micronucleus formation

**FIGURE 6:**
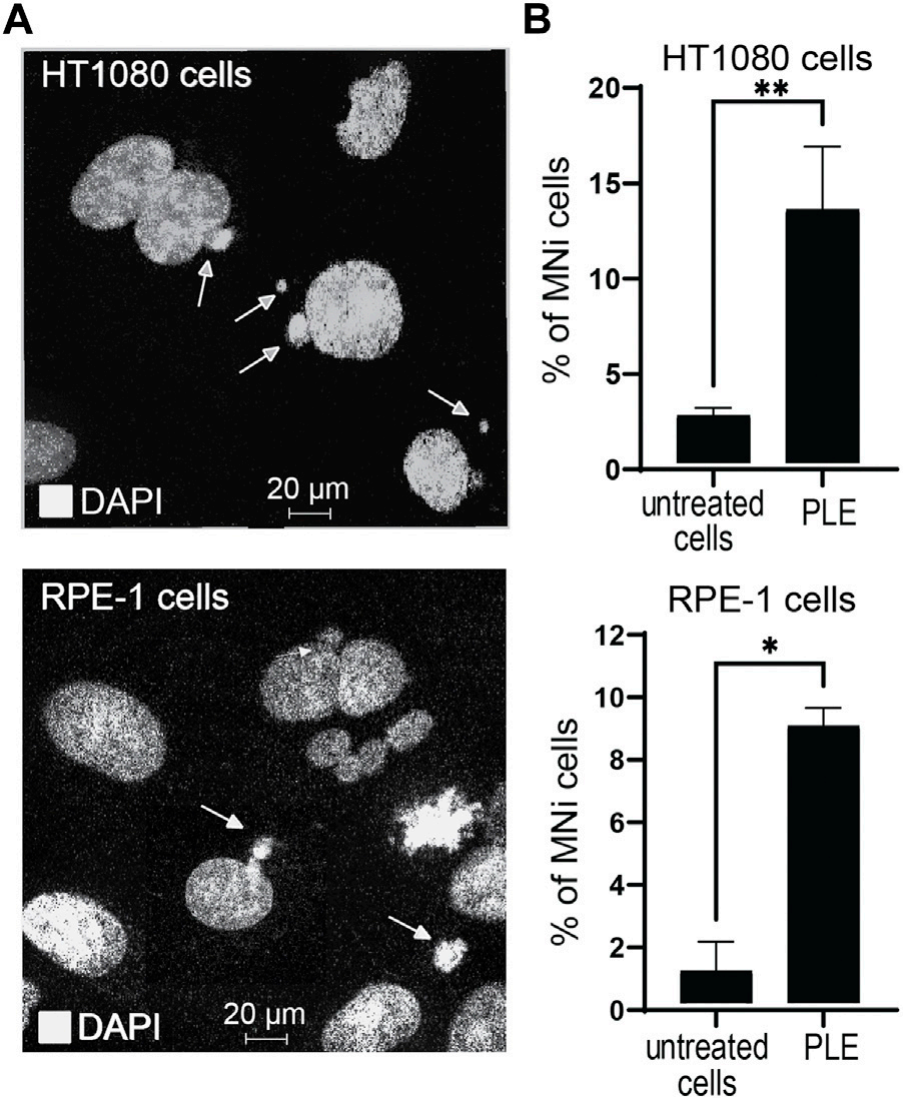
Micronuclei (MNi) formation 24 h after PLE treatment of HT1080 and RPE-1cells. **(A)** Examples of (MNi) formation in HT1080 and RPE-1 cells. White arrows point to the MNi. **(B)** The percentage of MNi after PLE treatment compared to untreated cells. Error bars correspond to a standard deviation (SD) of four replicates. Statistical analysis was performed using one-way ANNOVA, asterisks indicate statistical significance (*p < 0.05, **p < 0.01, relative to the negative control—untreated cells).

An micronucleus formation assay (MNi) has been extensively used to evaluate chromosome instability (Kirsch-Volders et al., 1997). To investigate whether PLE treatment leads to instability of the natural chromosomes, we performed an MNi assay in HT1080 and RPE-1 cells. Concentration of the PLE used in this experiment is presented in Table 1. Our analysis revealed a statistically significant elevation of MNi formation in both cells treated with PLE versus untreated (DMSO-treated negative control cells) (Figure 6A, Supplementary Figure S5). Data in Figure 6B are expressed in terms of percentages of the cells with MNi observed over the total number of binucleated cells analyzed and in terms of fold induction comparing treated versus untreated samples (Supplementary Table S3). Elevated frequencies of binucleated cells with MNi were observed in both HT1080 and RPE-1 cells. The percentage of MNi formation upon PLE treatment in HT1080 cells was 13.3 (SD ± 3.3; 5.3-fold elevation) and in RPE-1 cells 8.8 (SD ± 0.58; 8.5-fold elevation) (Figure 6B).

To summarize, MNi analysis confirmed that PLE has a significant effect on the accuracy of mitotic transmission of the natural chromosomes. The elevated frequencies of binucleated cells with MNi support our assumption that the PLE extract has the potential to be used as a CIN inducing drug.

### *P. granatum* leaf extract treatment induces formation of nucleoplasmic bridges

**FIGURE 7:**
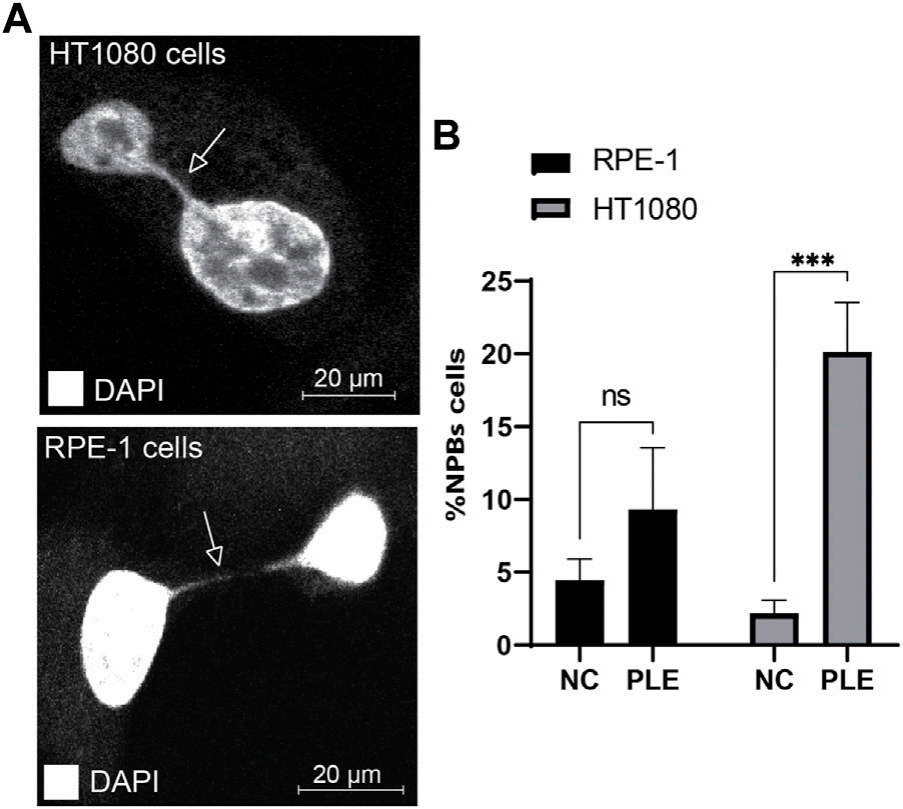
Accumulation of NPBs in HT1080 and RPE-1 cells treated with PLE. **(A)** Representative pictures of NPBs in HT1080 and RPE-1 cells after PLE treatment. **(B)** The percentage of NPBs in HT1080 and RPE-1 cells. Error bars correspond to a standard deviation (SD) of four replicates. Statistical analysis was performed using 2way ANNOVA, asterisks indicate statistical significance (*p < 0.005, ***p < 0.0005, relative to the negative control—untreated cells).

Nucleoplasmic bridges are a sensitive measure of chromosome damage leading to chromosome instability. Therefore, we determined formation of NPBs after PLE treatment. Concentration of the PLE extract used in this experiment is presented in Table 1. Our analysis revealed a statistically significant elevation of NPBs formation in HT1080 and RPE-1 cells treated with PLE versus untreated cells (Figure 7; Supplementary Figure S5). Data in Figure 7B are expressed in terms of percentages of the cells with NPBs observed over the total number of binucleated cells analyzed and in terms of fold induction comparing treated versus untreated samples. The percentage of NPBs formation in HT1080 cells was 27% (SD ± 5.7; 2.8-fold elevation). The percentage of NPBs formation in RPE-1 cells was 14% (SD ± 3.7; 1.75-fold elevation) (Supplementary Table S4).

Thus, the elevated frequencies of binucleated cells with MNi and NPBs (measures of genome damage and chromosomal instability) observed after PLE treatment strongly support the utility of the HAC/dGFP-based assay for screening new drugs.

### *P. granatum* leaf extract treatment leads to an increased number of double-stranded breaks

**FIGURE 8:**
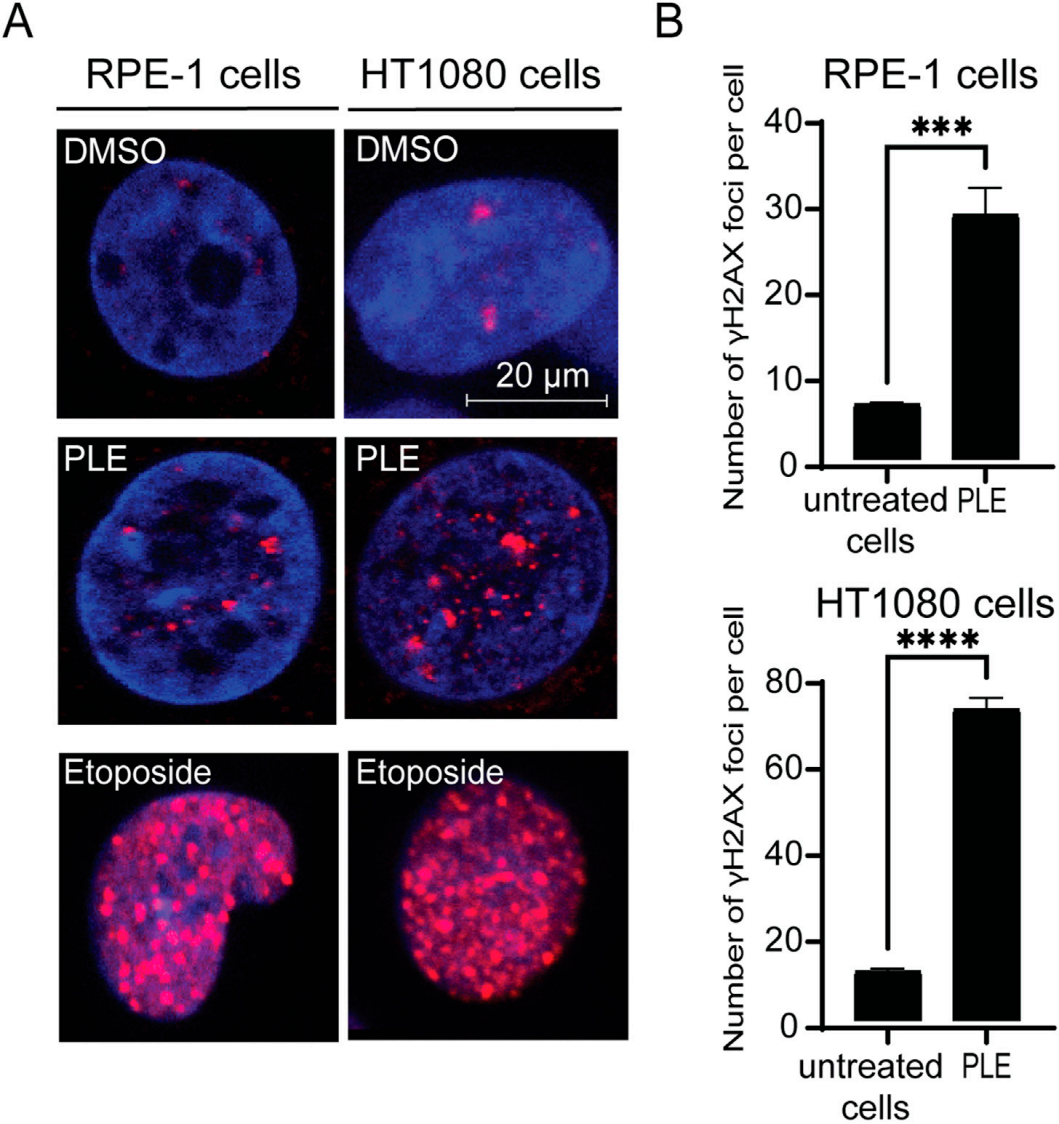
Accumulation of γH2AX foci in HT1080 and RPE-1 cells treated with PLE. **(A)** γH2AX immunostaining of HT1080 cells before and after treatment with PLE. **(B)** The percentage of γH2AX foci in HT1080 and RPE-1 cells. As a positive control, we used Etoposide – a drug commonly used for cancer therapy. Etoposide is an inhibitor of topoisomerase II that forms DSBs (Tassanee Lerksuthirat et all., 2020). Error bars correspond to a standard deviation (SD) of four replicates. Statistical analysis was performed using 2way ANNOVA, asterisks indicate statistical significance (***p < 0.001, ****p < 0.0001, relative to the negative control—untreated cells).

The cell cycle delay in G2 phase as well as reduction of mitosis, MNi and NPBs formation indicate possible DNA damage in the PLE-treated cells. Therefore, to investigate further the mechanism(s) by which treatment with PLE leads to chromosome instability, we stained HT1080 and RPE-1cells with an antibody against phosphorylated histone γH2AX, a common marker for DNA damage response (Mah et al., 2010), to detect formation of DNA double-stranded breaks (DSBs). The number of DSBs were expressed in terms of the number of γH2AX foci per nucleus and in terms of fold induction comparing treated versus untreated samples. Concentration of PLE used in this experiment is presented in Table 1. A statistically significant increase of γH2AX foci in interphase of the PLE-treated cells was observed in both HT1080 and RPE-1 cells (Figure 8; Supplementary Figure S5). The number of foci per cell in RPE-1 cells was 28 (SD ± 3.1; 4.3-fold elevation). In HT1080 cells, the number of foci was even higher and was equal to 72 (SD ± 2.6; 6.25-fold elevation) (Supplementary Table S6). Thus, in RPE-1 as well as in HT080 cells, chromosome instability after PLE treatment is accompanied by induction of DSBs.

To summarize, PLE treatment mediates cell cycle delay in G2/M phase and leads to reduction of mitosis, increased MNi, NPBs and γH2AX foci formation, indicating that *P. granatum* leaf extract contains some substances that elevate CIN. These data indicate a high potential of *P. granatum* extract as a source of anticancer compounds.

## Discussion

### A human artificial chromosomes/dGFP-based system for identifying novel chromosome instability inducing compounds

A wide range of methods is applicable for measuring chromosome instability (CIN). The rate of chromosome mis-segregation may be quantified by coupling clonal cell analysis with karyotyping or fluorescence *in situ* hybridization (FISH) (Kirsch-Volders et al., 1997; Bakker et al., 2015; Thompson and McManus, 2015). The most widely used method is the micronuclei formation assay (Kirsch-Volders et al., 1997), which allows to extrapolate the frequency of MNi formation to the rate of chromosome loss. It is worth noting that an MNi assay does not measure the fraction of drug-arrested cells that undergo mitosis and form viable aneuploid cells. Till now a karyotype analysis and a micronuclei assay remain commonly used to study CIN and CIN induction by anticancer drugs and environmental agents.

Recently the HAC-based technology has been developed for drugs screening to manipulate the CIN phenotype in cancer cells (Lee et al., 2013; Kim et al., 2016; Lee et al., 2016). There are several advantages of the HAC-based assays compared to karyotype and MNi methods. Firstly, the HAC-based assays are significantly faster and less labor-intensive. Secondly, microplate reader, as well as flow cytometry, can rapidly and simultaneously analyze thousands of cells compared to other methods. Finally, the HACs contain a functional centromere that provides their efficient and proper transfer through mitosis. At the same time the HACs have a relatively small size that varies from 1 to 10 Mb. For example, the alphoid^tetO^-HAC is approximately 5 Mb in size (Kouprina et al., 2012) that results in its spontaneous loss (per cell division) roughly 10-fold higher than that of native chromosomes (Nakano et al., 2008; Lee et al., 2013), making the alphoid^tetO^-HAC-based assay very sensitive for measuring CIN. Together, these HAC features allow detection of even small differences between the rates of chromosome loss induced by the chemical compounds affecting chromosome stability (Lee et al., 2016). This is important because an accurate assessment of CIN is crucial to select the drugs with the highest effect on chromosome transmission.

In this study, the HAC/dGFP-based system, previously developed for screening different siRNA libraries to identify the genes involved in CIN, was adapted to screen and identify anticancer drugs that selectively affect chromosome segregation. We believe that our HAC-based assay will become the method of choice to find new anticancer drugs that induce tumor cells death through elevation of their chromosomal instability.

### *P. granatum* leaves extract elevates chromosome instability in cancer cells

It is well established that endemic plant secondary metabolites have pluripharmacologic properties inclusive of antiproliferative activities, as corroborated by several *in vitro* and *in vivo* studies (Musial et al., 2020; Vafadar et al., 2020). Betulinic acid, a triterpene, is exploited for its antiproliferative activity against a broad spectrum of cancer cell types with a limited adverse effect on non-malignant cells (Przystupski et al., 2019). Earlier works by Neergheen et al. (Neergheen et al., 2006; Neergheen et al., 2007; Neergheen et al., 2011), Soobrattee et al. (2008), Ramhit et al. (2018), Mahomoodally et al. (2019), and Rummun et al. (Rummun et al., 2019; Rummun et al., 2020; Rummun et al., 2021a; Rummun et al., 2021b) have reported the *in vitro* antiproliferative effect of the plants used in this study (Table 1). Though the examples of the natural products, which anticancer properties were highly rated and medicinally exploited, are abundant in the literature (Freiburghaus et al., 1996; Costa-Lotufo et al., 2005; Cai et al., 2006; Fouche et al., 2008; Kamatou et al., 2008; Ochwang'i et al., 2014) an intensive search for the potent antitumor agents still remains.

In this study, we demonstrated for the first time the direct CIN induction by the pomegranate leaves extract. Pomegranate (*P. granatum*) has been used for centuries in the traditional medicine of different countries as a source of diverse bioactive compounds with a proven intriguing pharmacological potential (Modaeinama et al., 2015). The *P. granatum* extracts show the difference in phenolic profiling and antioxidant capacity in flowers, seeds, leaves, and peels (Fellah et al., 2018). The *P. granatum* leaves extract contains gallic acid, ellagic acid, gallocatechin, delphinidin, cyanidin, pelargonidin, and sitosterol, which are not only powerful antioxidants but were suggested to be attractive sources for discovery of anticancer compounds (Supplementary Table S7) (Nawwar et al., 1994; Lei et al., 2003). Extensive research over the past few years has revealed that the extracts derived from various organs of the pomegranate plant have the direct antineoplastic potential (Khwairakpam et al., 2018). It was detected that the pomegranate flower extract (PFE) stimulates apoptosis and antiproliferative activity of U266 and B16f10 cells (Kiraz et al., 2016; Sharifiyan et al., 2020). The pomegranate peel extract (PPE) significantly reduces viability of numerous cancer cell lines even at the lowest concentration with no or less visible toxicity in normal cells. It was also detected that PPE is responsible for arrest of melanoma cells in G2/M phase and G1 delay of colon carcinoma cells (Modaeinama et al., 2015). Intriguingly, pomegranate peel dietary intake reduces total chromosomal aberrations and aberrant metaphase frequencies of barium-treated rats (Elwej et al., 2016). MTT assay showed the cytotoxicity and antiproliferation effect of the pomegranate seed oil in esophageal, breast, and ovarian cancer cell lines (Shirode et al., 2014; Seidi et al., 2016; Zare et al., 2021).

While many previous studies have shown an antioxidant and tumor-suppressive effect of extracts from the peel, seeds, and steam, in the current study we observed the ability of *P. granatum* leaves extract (PLE) to elevate chromosome instability. This is in agreement with some studies where it was shown a potential tumor suppressive property of PLE. More specifically, PLE inhibits cell proliferation in the non-small cell lung carcinoma cell line and affects H1299 cell survival by arresting cell cycle progression in G2/M phase in a dose-dependent manner and inducing apoptosis (Li et al., 2016). In other studies, it was shown that PLE contains abundant polyphenols, mainly gallotannins, ellagitannins, and ellagic acid (Sreekumar et al., 2014; Alberto Coronado-Reyes et al., 2021), that was found to interfere with proliferation, cell cycle progression, and apoptosis *in vitro* and *in vivo* (Kiraz et al., 2016; Tibullo et al., 2016; Sharifiyan et al., 2020).

While our data strongly support inhibition of cell proliferation (Figure 3) and cell cycle arrest (Figure 4), none of the previous studies has shown the dynamic progression of cell cycle after PLE treatment. Our data demonstrated that recovery from PLE treatment results in disruption of cell cycle progression such as G2/M arrest (Figure 4). The G2/M checkpoint allows to evaluate whether the processes at the previous phases of cell cycle have been properly completed. The G2/M checkpoint is basically influenced by incorrect DNA replication and damage (Wenzel and Singh, 2018). DNA damage induction is a common strategy for cancer chemotherapy. Many DNA damage-inducing drugs utilize DSBs formation, such as etoposide, teniposide, doxorubicin, daunorubicin, idarubicin, and mitoxantrone. In this study, we used Etoposide as a positive control because, as known, it affects DSBs (Figure 8). We observed the comparable effect with PLE and Etoposide treatment that allows to assume that one of the PLE metabolites causes CIN through DNA damage.

As known, DNA damage induces a high level of transient protein-associated breaks in the genome of treated cells. The potential lethality of DSBs rises dramatically when replication machinery or helicases attempt to work on the damaged DNA template. This disrupts the cleavage complex and converts transient single- or double-strand breaks into the permanent double-stranded leisure which is no longer held together by proteinaceous bridges. These breaks may become the targets for recombination, sister chromatid exchange, generation of large insertions and deletions, and ultimately the production of chromosomal aberrations.

Another study demonstrated cell cycle arrest in G2/M after treatment with the pomegranate extract (PE) from fruit skins (Shirode et al., 2014). The authors performed a DNA microarray analysis that revealed that PE downregulates the genes associated with mitosis, chromosome organization, RNA processing, DNA replication, and DNA repair, and upregulates the genes involved in regulation of apoptosis and cell proliferation. Both microarray and quantitative RT-PCR indicated that PE downregulates the genes involved in DNA double strand break repair by homologous recombination (HR), such as MRE11, RAD50, NBS1, RAD51, BRCA1, BRCA2, and BRCC3. Downregulation of HR genes correlates with the increased level of their predicted microRNAs (miRNAs), miR-183 (predicted target RAD50), and miR-24 (predicted target BRCA1), suggesting that PE may regulate miRNAs involved in DNA repair processes. Further, PE treatment increases the frequency of DSBs. All these observations suggest that PE downregulates HR which sensitizes the cells to DSBs, growth inhibition, and apoptosis (Shirode et al., 2014). These data support the conclusion that the *P. granatum* leave extract induces CIN through DSBs formation. However, the mechanism underlying CIN formation after PLE treatment must be studied by utilizing pure components of the extract.

Our data revealed a significant increase in mitotic abnormalities in PLE-treated cancer cells that included defects in chromosome alignment and multipolar spindle formation (Figure 5). Failure in these processes leads to chromosome mis-segregation that results in abnormal numbers of chromosomes, known as aneuploidy. Identification a specific compound of PLE will allow us to investigate the mechanism(s) by which treatment by this compound results in mitotic abnormalities.

To summarize, the HAC/dGFP-based assay used in this study can be utilized for a further thorough analysis of the purified *P. granatum* metabolites, including ellagic acid, gallic acid, gallocatechin, delphinidin, cyanidin, pelargonidin, and sitosterol to pick up the individual substance responsible for MNi and NPBs formation and DSBs. Out of a wide range of the methods applied to measure CIN, the HAC/dGFP system allows to measure CIN quantitatively and may serve as an effective biotechnological tool for high-throughput screening of new candidate drugs inducing a CIN level over the survival threshold for cancer cells.

### In Memoriam

In loving memory of Dr. Alexander Kagansky who passed away on 21 December 2020.

## Data Availability

The raw data supporting the conclusion of this article will be made available by the authors, without undue reservation.
