# Supplementary material for: Novel assay to measure chromosome instability identifies Punica granatum extract that elevates CIN and has a potential for tumor- suppressing therapies
Source: Front Bioeng Biotechnol. 2022 Dec 19;10:989932. doi: 10.3389/fbioe.2022.989932 (PMC9806258; doi:10.3389/fbioe.2022.989932)
Supplement: Supplementary file 1 [file Table1.DOCX]

**Supplemental Material**

for

**Novel Assay to Measure Chromosome Instability Identifies *Punica granatum* Extract that Elevates CIN and has a Potential for Tumor- Suppressing Therapies**

Nikolay V. Goncharov^1,4*^, Valeria A. Kovalskaia^2^, Alexander O. Romanishin^3^, Nikita A. Shved ^1,4^, Andrei S. Belousov^4^, Vladlena S. Tiasto^4^, Valeria S. Gulaia^4^, Vidushi S. Neergheen^5^, Vladimir Larionov^6^, Natalay Kouprina^6^ and Vadim V. Kumeiko^1,4^

^1^A.V. Zhirmunsky National Scientific Center of Marine Biology, Far Eastern Branch, Russian Academy of Sciences, 690041 Vladivostok, Russia

^2^Research Centre for Medical Genetics, Moscow, 115522, Russia

^3^School of Life Sciences, Immanuel Kant Baltic Federal University, Kaliningrad, 236041, Russia

^4^Institute of Life Sciences and Biomedicine, Far Eastern Federal University, Vladivostok, 690922, Russia

^5^ Biopharmaceutical Unit, Centre for Biomedical and Biomaterials Research (CBBR), University of Mauritius, R[éduit, Moka](https://en.wikipedia.org/wiki/R%C3%A9duit,_Moka), Mauritius

^6^Developmental Therapeutics Branch, National Cancer Institute, NIH, Bethesda, MD 20892, USA

Corresponding Author
[goncharovnv.gn@gmai.com](mailto:goncharovnv.gn@gmai.com)

**Content:**

1. Supplemental Figures S1-S5
2. Supplemental Tables S1-S7
3. Supplemental Methods

*
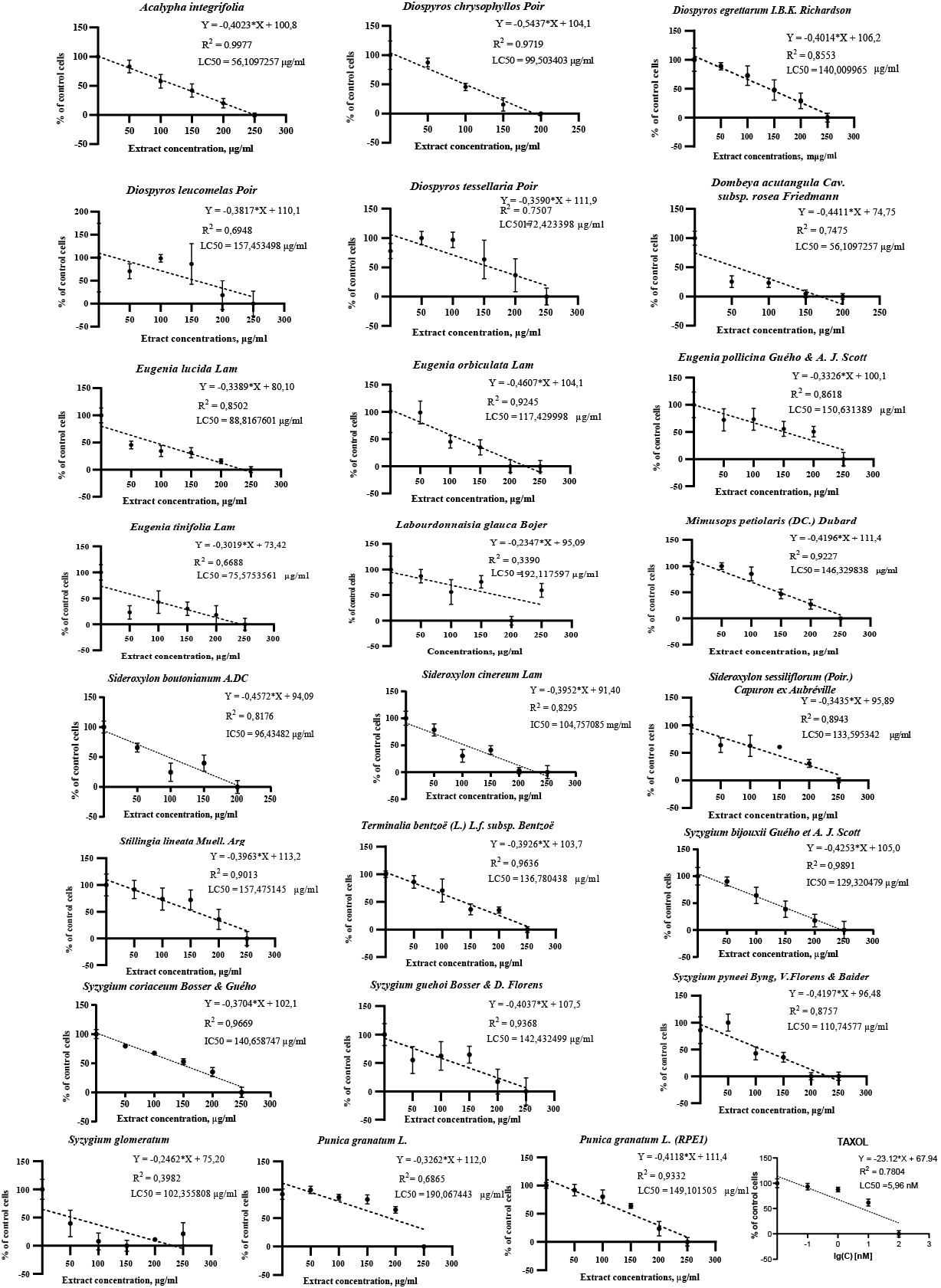
*

**Figure S1** The effect of the extracts on cell viability after 24 hrs of treatment. For each extract, linear regression analysis was performed showing Mean ± SD and trend line, alongside representing the R^2^, trend line equation, and LC_50_.

# *
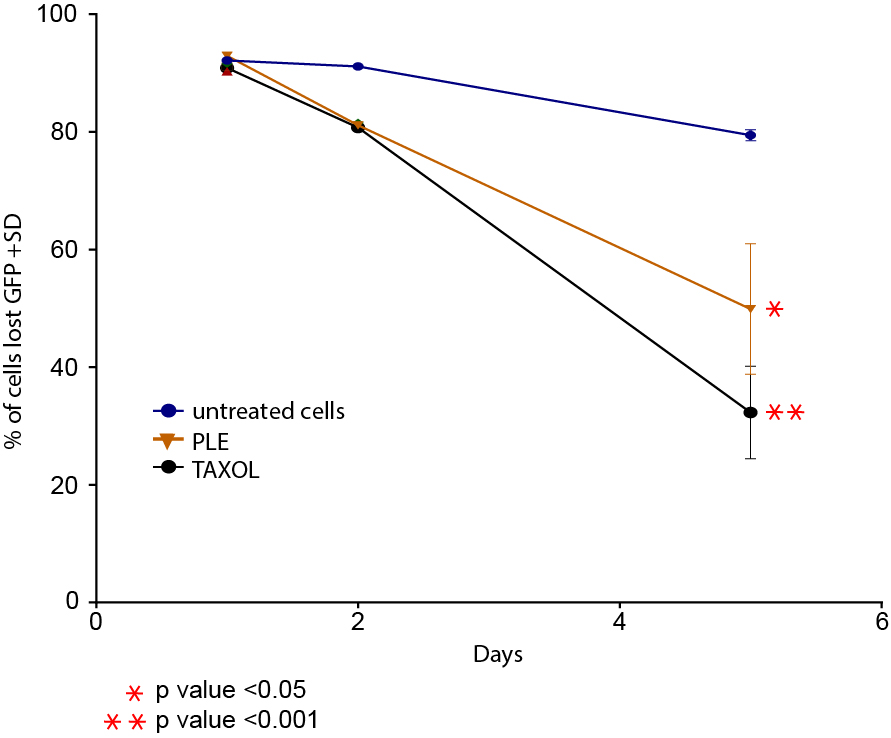
*

**Figure S2** The actual percentage of HAC/dGFP loss upon PLE treatment measured by FACS after 2, 4 and 6 days. Taxol treatment was used as a positive control. Untreated cells were used as a negative control. PLE was added to the cells at the concentration LC_50_ and treated for 24 hrs. Red asterisks indicate statistical significance (p<0.005) in comparison with a negative control.

**
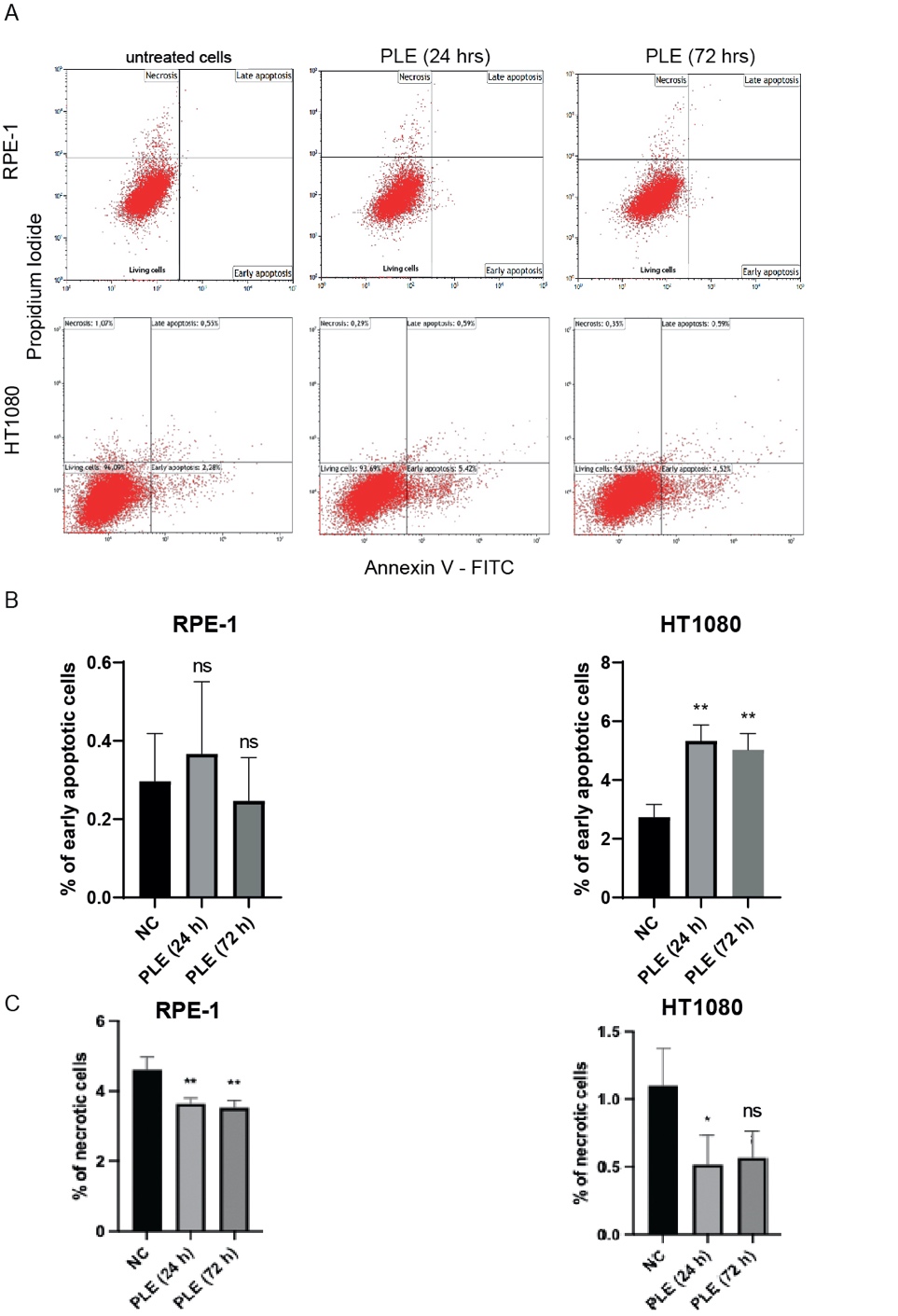
**

**Figure S3** Analysis of apoptosis after PLE treatment. (**A**) Representation of flow cytometry analysis of HT1080 and RPE-1 cells 24 and 72 hrs after PLE treatment. **(B**) Comparison of early apoptotic and necrotic (**C**) cells 24 and 72 hrs after PLE treatment. The statistical analysis was performed using parametric One-way ANOVA (Dunnet correction), asterisks indicate statistical significance (** p<0.01, ns - non-significant, relative to untreated cells).


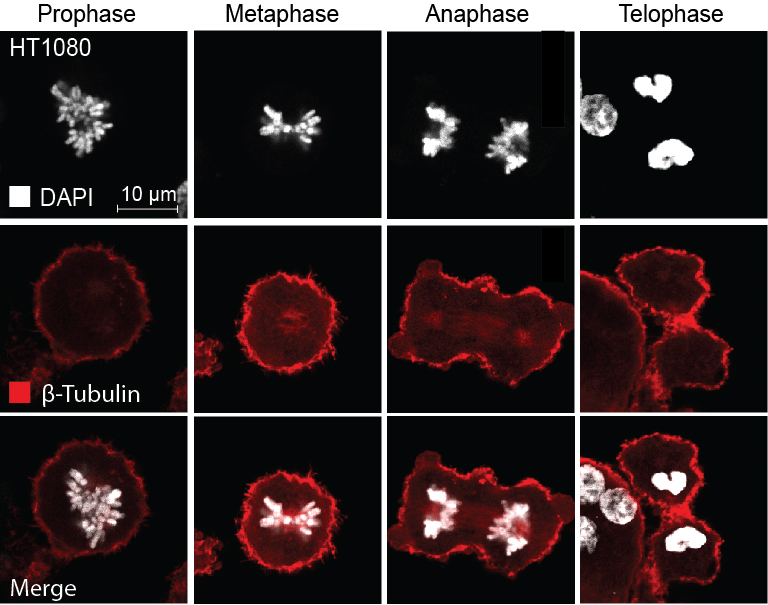


**Figure S4** Representative pictures of mitotic stages in HT1080 cells. Localization of tubulin beta at the different stages of mitosis in untreated HT1080 cells. Staining by antibodies against tubulin beta is marked in red, DAPI in white.


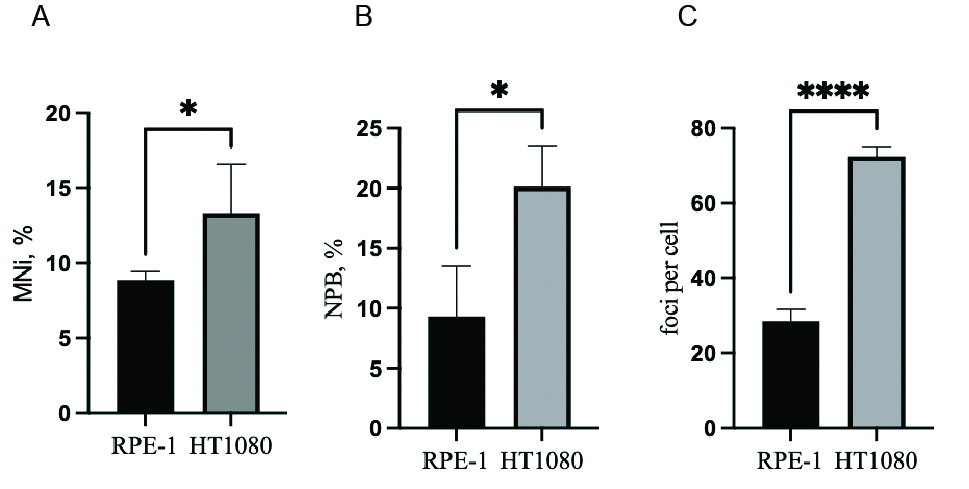


**Figure S5** Comparison between HT1080 and RPE-1 cells treated with PLE. (**A**) Micronuclei (MNi) formation 24 hrs after PLE treatment of HT1080 vs. RPE-1cells. (**B**) Accumulation of nucleoplasmic bridges (NPBs) in HT1080 vs. RPE-1 cells treated with PLE. (**C**) Accumulation of γH2AX foci in HT1080 vs. RPE-1 cells treated with PLE. Comparison between HT1080 and RPE-1 cells post PLE treatment was done by one-tailed unpaired t-test.

**Table S1** Cell cycle analysis of HT1080 and RPE-1 cells after PLE treatment. Average percentages, standard deviations (± SD), fold changes compared to the control and p values of 2-way Anova test with Sidak multiple comparisons [fold changes and p values were calculated when compare the sample (PLE treated) and untreated cells (DMSO)]

| Exposure time, hrs | Cells | Phase | Extract | % of cells in cell cycle phases | ± SD | Fold increase | p value |
| --- | --- | --- | --- | --- | --- | --- | --- |
| 24 | HT1080 | G1 | DMSO | 65.11 | 1.4 | 1.46 | <0.0001 |
|  |  |  | PLE | 95.28 | 1.42 |  |  |
|  | RPE-1 | S | DMSO | 11.88 | 0.49 | 1.59 | <0.0001 |
|  |  |  | PLE | 18.89 | 0.36 |  |  |
|  |  | G2/M | DMSO | 18.55 | 0.49 | 1.27 | <0.0001 |
|  |  |  | PLE | 23.54 | 0.22 |  |  |
| 72 | HT1080 | G2/M | DMSO | 12.93 | 1.7 | 2.72 | <0.0001 |
|  |  |  | PLE | 35.2 | 0.4 |  |  |
|  | RPE-1 | S | DMSO | 11.07 | 0.5 | 1.73 | <0.0001 |
|  |  |  | PLE | 19.13 | 2.32 |  |  |
|  |  | G2/M | DMSO | 17.65 | 0.32 | 1.74 | <0.0001 |
|  |  |  | PLE | 30.65 | 0.83 |  |  |

**Table S2** The rates of mitotic phenotypes in the mitotic index assay after PLE treatment of HT1080 and RPE-1 cells in comparison with vehicle (DMSO) treatment. Average percentages, standard deviations (± SD), fold change compared to the control and p values of 2-way Anova test with Sidak multiple comparisons

| Exposure time,  hrs | Cells | Number of cells evaluated in mitotic index assay | Treatment | Mitotic index (average) % | ± SD | Fold decrease | p value |
| --- | --- | --- | --- | --- | --- | --- | --- |
| 24 | RPE-1 | 1028 | DMSO | 4.358 | 0.8148 | 10.91 | 0.0025 |
|  |  | 980 | PLE | 0.3992 | 0.1931 |  |  |
|  | HT1080 | 975 | DMSO | 6.020 | 0.5320 | 8.21 | 0.0008 |
|  |  | 990 | PLE | 0.7329 | 0.2587 |  |  |
| 72 | RPE-1 | 965 | DMSO | 4.281 | 0.3932 | 0.92 | 0.2036 |
|  |  | 825 | PLE | 4.643 | 0.07290 |  |  |
|  | HT1080 | 984 | DMSO | 6.446 | 0.1780 | 2.05 | <0.0001 |
|  |  | 1103 | PLE | 3.141 | 0.1615 |  |  |

The data set was obtained by the average of four independent experiments for the PLE compound.

**Table S3** The numbers of micronuclei (MNi) formations in HT1080 and RPE-1 cells after PLE treatment. Average percentages, standard deviations (± SD), fold changes compared to the control and p values of 2-way Anova test with Sidak multiple comparisons [fold changes and p values are calculated when compare the sample (PLE-treated) and untreated cells (DMSO)]

| Cells | Number of cells evaluated in micronucleation assay | Treatment | Number of MNi (average) % | ± SD | Fold increase | p value |
| --- | --- | --- | --- | --- | --- | --- |
| RPE-1 | 450 | DMSO | 1.03 | 0.93 | 8.5 | 0.0133 |
|  |  | PLE | 8.8 | 0.58 |  |  |
| HT1080 | 450 | DMSO | 2.5 | 0.37 | 5.3 | 0.0041 |
|  |  | PLE | 13.3 | 3.3 |  |  |

**Table S4** The numbers of nucleoplasmic bridges (NPBs) in HT1080 and RPE-1 cells after PLE treatment. Average percentages, standard deviations (± SD), fold changes compared to the control and p values of 2-way Anova test with Sidak multiple comparisons [fold changes and p values are calculated when compare the sample (PLE-treated) and untreated cells (DMSO)]

| Cells | Number of cells evaluated in NPB assay | Number of NPBs | Treatment | NPBs (average) % | ± SD | Fold increase | p value |
| --- | --- | --- | --- | --- | --- | --- | --- |
| RPE-1 | 73 | 2 | DMSO | 4.457 | 1.448 | 2.094 | 0.1319 |
|  | 114 | 6 |  |  |  |  |  |
|  | 84 | 4 |  |  |  |  |  |
|  | 68 | 5 | PLE | 9.333 | 4.209 |  |  |
|  | 106 | 6 |  |  |  |  |  |
|  | 73 | 9 |  |  |  |  |  |
| HT1080 | 134 | 2 | DMSO | 2.178 | 0.8981 | 9.247 | 0.0001 |
|  | 129 | 4 |  |  |  |  |  |
|  | 112 | 2 |  |  |  |  |  |
|  | 87 | 16 | PLE | 20.14 | 3.396 |  |  |
|  | 90 | 16 |  |  |  |  |  |
|  | 93 | 13 |  |  |  |  |  |

**Table S5** The numbers of mitotic abnormalities in HT1080 cells after PLE treatment. Average percentages, standard deviations (± SD), fold changes compared to the control and p values of 2-way Anova test with Sidak multiple comparisons [fold changes and p values are calculated when compare the sample (PLE-treated) and untreated cells (DMSO)]

| Cells | Number of cells evaluated in the assay | Number of found abnormalities | Treatment | Number of abnormalities  (average) % | ± SD | Fold increase | p value |
| --- | --- | --- | --- | --- | --- | --- | --- |
| HT1080 | 133 | 3 | DMSO | 1.451 | 0.7464 | 3.8745 | 0.0029 |
|  | 152 | 2 |  |  |  |  |  |
|  | 128 | 1 |  |  |  |  |  |
|  | 150 | 7 | PLE | 5.622 | 0.8278 |  |  |
|  | 147 | 9 |  |  |  |  |  |
|  | 181 | 11 |  |  |  |  |  |

**Table S6** The quantity of γH2AX foci in HT1080 and RPE-1 cells after PLE treatment compared to vehicle (DMSO) treatment. Average percentages, standard deviations (± SD), fold changes in comparison to the control and p values of 2-way Anova test with Sidak multiple comparisons [fold changes and p values are calculated when compare the sample (PLE-treated) and untreated cells (DMSO)]

| Cells | Number of cells evaluated in DNA damage assay | Treatment | γH2AX foci number per cell (average)% | ± SD | Fold increase | p value |
| --- | --- | --- | --- | --- | --- | --- |
| RPE-1 | 65 | DMSO | 6.5 | 0.1498 | 4.3 | 0.0003 |
|  | 65 | PLE | 28 | 3.137 |  |  |
| HT1080 | 65 | DMSO | 11.52 | 0.6094 | 6.25 | <0.0001 |
|  | 65 | PLE | 72 | 2.6 |  |  |

The data set was obtained by the average of three independent experiments for the PLE compound.

**Table S7** Pomegranate components

| № | Pomegranate Phytochemicals | Formula | Molecular weight (MW) | Plant Part |
| --- | --- | --- | --- | --- |
| **Ellagitannins and Gallotannins** | | | | |
| 1 | Corilagin | C27H22O18 | 634.45 | Fruit, leaves, pericarp |
| 2 | Cyclic 2,4:3,6-bis(4,4′,5,5′,6,6′-hexahydroxy [1,1′-biphenyl]- 2,2′-dicarboxylate) 1-(3,4,5-trihydroxybenzoate) b-D-Glucose | C41H28O26 | 936.65 | leaves |
| 3 | Punicafolin | C41H30O26 | 938.66 | Leaves |
| 4 | Strictinin | C27H22O18 | 634.45 | Leaves |
| 5 | Tellimagrandin I | C34H26O22 | 786.56 | Leaves, pericarp |
| 6 | Tercatain | C34H26O22 | 786.56 | Leaves |
| 7 | 5-O-galloyl-punicacortein D | C54H34O34 | 1222.8 | Leaves |
| **Ellagic Acid Derivatives** | | | | |
|  | Ellagic acid | C14H6O8 | 302.19 | Fruit, pericarp, bark |
|  | Ellagic acid, 3,3′-di-O-methyl | C16H10O8 | 330.25 | Seed |
|  | Ellagic acid, 3,3′, 4′-tri-O-methyl | C17H12O8 | 344.27 | Seed |
|  | Ellagic acid, 3′-O-methyl-3, 4-methylene | C16H8O8 | 328.23 | Heartwood |
|  | Eschweilenol C | C20H16O12 | 448.33 | Heartwood |
| **Anthocyanins and Anthocyanidins** | | | | |
| 8 | Apigenin-4′-O-*β*-D-glucoside | C21H20O11 | 448.32 | Leaves |
| 9 | Luteolin-3′-O-*β*-D-glucoside | C21H20O10 | 432.11 | Leaves |
| 10 | Luteolin-4′-O-*β*-D-glucoside | C21H20O10 | 432.11 | Leaves |
| 11 | Luteolin-3′-O-*β*-D-Xyloside | C21H18O10 | 418.09 | Leaves |
| 12 | Eriodictyol-7-O-*α*-Larabinofuranosyl (1-6)-*β*-D-glucoside | C26H30O15 | 582.51 | Leaves |
| 13 | Naringenin 4′-methylether 7-O-*α*-L-arabinofuranosyl (1-6)-*β*-D-glucoside | C27H32O14 | 580.53 | Leaves |
| 14 | Brevifolin | C12H8O6 | 248.19 | Leaves |
| 15 | Brevifolin carboxylic acid | C13H8O8 | 292.2 | Leaves |
| 16 | Brevifolin carboxylic acid-10-monosulphate | C13H7KO10S | 394.25 | Leaves |
| 17 | 1,2,3-Tri-O-galloyl-*β*-D-glucose | C27H24O18 | 448.32 | Leaves |
| 18 | 1,2,4-Tri-O-galloyl-*β*-D-glucose | C27H24O18 | 286.24 | Leaves |
| 19 | 1,2,6-Tri-O-galloyl-*β*-D-glucose | C27H24O18 | 286.24 | Leaves |
| 20 | 1,4,6-Tri-O-galloyl-*β*-D-glucose | C27H24O18 | 432.11 | Leaves |
| 21 | 1,3,4-Tri-O-galloyl-*β*-D-glucose | C27H24O18 | 432.11 | Leaves |
| 22 | 1,2, 4, 6-Tetra-O-galloyl-*β*-D-glucose | C34H28O22 | 418.09 | Leaves |
| 23 | 1,2,3,4, 6-Pent-O-galloyl-*β*-D-glucose | C41H32O26 | 318.04 | Leaves |
| 24 | 3,4,8,9,10-pentahydroxy-dibenzo[b,d]pyran-6-one | C13H8O7 | 464.38 | Leaves |
| 25 | *β*-Sitosterol | C29H50O | 414.71 | Seed oil, leaves, stem |
| 26 | 1-(2,5-dyihydroxy-phenyl)-pyridium chloride | C11H10ClNO2 | 223.66 | Leaves |

**MATERIALS AND METHODS**

**Calculation of the rate of spontaneous HAC loss and after extract treatment**

To calculate the rate of HAC loss after cell treatment by an extract, we used the formula R= 2-2(P_n_/P_0_)^(1/n)^ where P_0_ is the percentage of HAC-containing cells in the population cultured under selection before drug treatment, P_n_ is the percentage of HAC-containing cells extract treatment in absence of selection, n is the number of cell doublings that occurs during treatments with extract and culturing without selection after the drug treatment.
